# Exploring glycerophospholipid metabolism in nasopharyngeal carcinoma: interactions between malignant epithelial cells and CCL11-expressing fibroblasts

**DOI:** 10.3389/fimmu.2026.1799551

**Published:** 2026-05-20

**Authors:** Liping Wang, Dujuan Wang, Shuang Li, Guohong Liu, Yirong Li, Yunbao Pan

**Affiliations:** 1 Department of Laboratory Medicine, Zhongnan Hospital of Wuhan University, Wuhan University, Wuhan, China; 2 Hubei Provincial Clinical Research Center for Molecular Diagnostics, Wuhan, China; 3 Department of Clinical Pathology, Houjie Hospital of Dongguan, The Affiliated Houjie Hospital of Guangdong Medical University, Dongguan, China; 4 Department of Otolaryngology Head and Neck Surgery, Zhongnan Hospital of Wuhan University, Wuhan, China; 5 Department of Radiology, Zhongnan Hospital of Wuhan University, Wuhan University, Wuhan, China

**Keywords:** glycerophospholipid metabolism, nasopharyngeal carcinoma, spatial metabolomics, spatial transcriptomics, tumor microenvironment

## Abstract

**Background:**

Nasopharyngeal carcinoma (NPC) is associated with aberrant cellular metabolism and interactions between tumor and stromal cells. This study aims to elucidate the role of glycerophospholipid metabolism in NPC, particularly focusing on the interplay between malignant epithelial cells and fibroblasts.

**Methods:**

We employed a multi-omics approach integrating single-cell transcriptomics, 10x spatial transcriptomics, and spatial metabolomics to analyze the gene expression and metabolite profiles in NPC tissues. Differential metabolite abundance and gene expression were evaluated to identify key glycerophospholipid-related genes.

**Results:**

We identified five glycerophospholipid-related genes—AGPAT3, DGAT2, SLC44A1, AGPAT5, and LPGAT1—that were significantly upregulated in NPC and EBER+ tumor-enriched regions. Fibroblasts expressing CCL11 were found to be associated with fatty acid accumulation and potentially with enhanced glycerophospholipid metabolism through interactions with malignant epithelial cells. Additionally, spatial trajectory analysis indicated shared and distinct gene expression patterns during differentiation toward peritumoral and intratumoral immune regions, revealing a complex landscape of NPC invasion.

**Conclusion:**

Our findings suggest that glycerophospholipid metabolism may play an important role in NPC progression and highlight the potential of AGPAT3, DGAT2, SLC44A1, AGPAT5, and LPGAT1 as diagnostic and prognostic markers. This study provides novel insights into the metabolic interactions within the NPC microenvironment, providing insights for potential targeted therapeutic interventions.

## Introduction

Nasopharyngeal carcinoma (NPC) is a malignant epithelial tumor located in the head and neck region, characterized by significant infiltration of immune cells. Its association with Epstein-Barr virus (EBV) infection and high prevalence in specific geographic areas, including Southeast Asia, North Africa, and southern China, underscores its clinical importance (1, 2). Due to its asymptomatic nature in the early stages, over 90% of NPC cases are diagnosed at advanced stages, where conventional treatments like chemoradiotherapy are often less effective for recurrent and metastatic disease (3). This highlights the urgent need for identifying biomarkers that can aid in early diagnosis, inform treatment decisions, and predict patient prognosis.

A critical challenge in managing advanced NPC is its biological heterogeneity (3). While bulk RNA sequencing provides a broad overview of gene expression in NPC tissues, it fails to capture cellular diversity, obscuring vital insights into the tumor microenvironment. In contrast, single-cell sequencing offers a more nuanced resolution, revealing the malignant characteristics of epithelial cells alongside the extensive infiltration of various immune cell types (4). The NPC microenvironment comprises diverse cellular populations, including T cells, B cells, natural killer (NK) cells, myeloid cells, endothelial cells, fibroblasts, normal epithelial cells, and malignant epithelial cells (5–7). However, both bulk and single-cell sequencing approaches disrupt tissue architecture, limiting our ability to accurately capture the spatial organization of cells within the tumor (8).

To address these limitations, spatial transcriptomics has emerged as a powerful tool that provides spatially resolved gene expression profiles within intact tissues, illuminating the spatial heterogeneity of the tumor microenvironment (9). Additionally, spatial mass spectrometry imaging (MSI) enables the direct mapping of metabolites within tissue sections, offering qualitative, quantitative, and locational insights into metabolite distribution (10, 11). MSI-based spatial metabolomics further characterizes the metabolic landscape of tumor tissues, providing a comprehensive view of spatial metabolic heterogeneity (12).

In this study, we integrate single-cell transcriptomics, 10x spatial transcriptomics, and spatial metabolomics to investigate the spatial distribution of gene expression and metabolites within the NPC microenvironment. We focus on the role of fibroblasts, their interactions with malignant epithelial cells, and the implications for metabolism. Additionally, we employ digital spatial profiling (DSP) to validate the prognostic value of metabolism-related genes. Our objective is to map differentiation trajectories and elucidate potential functional roles across distinct regions of NPC at high spatial resolution. By identifying biomarkers at the single-cell spatial level, this study aims to provide a theoretical foundation for developing targeted therapies, paving the way for personalized treatment strategies for NPC patients.

## Materials and methods

### Sample collection

We collected 20 untreated samples (15 nasopharyngeal carcinoma (NPC) and 5 nasopharyngeal chronic inflammation with hyperplasia (CIH)) at Zhongnan Hospital of Wuhan University for multi-omics studies. Of these, 9 samples underwent single-cell transcriptome sequencing, 8 samples were processed for 10x spatial transcriptomics, and 16 samples were used for spatial metabolomics analysis. These samples as well as the spatial transcriptomics and spatial metabolomics analytical workflows applied in this study have been previously established and reported in our published studies (12). All participants provided informed consent, and the study was approved by the Ethics Committee of Zhongnan Hospital of Wuhan University (2022206K). All methods were carried out in accordance with relevant guidelines and regulations. Additionally, 40 tumor-enriched regions from previously collected DSP spatial transcriptomics data (13) were selected for survival prognosis analysis. To validate gene expression differences between NPC and normal tissues, we downloaded mRNA expression data for 41 samples (31 NPC and 10 normal nasopharyngeal tissues) from the GEO database (GSE12452).

### Detection of EBV infection by EBER *In Situ* hybridization

EBV infection status was determined by detecting Epstein-Barr virus-encoded small RNAs (EBERs). In this study, EBER *in situ* hybridization (ISH) was performed on formalin-fixed, paraffin-embedded (FFPE) tissue specimens using a commercially available EBER ISH kit (Roche Diagnostics, Shanghai, China), strictly following the manufacturer’s instructions. 4 μm-thick tissue sections were prepared and subjected to routine deparaffinization and rehydration, followed by pretreatment. Proteinase digestion was then carried out to adequately expose the target nucleic acid sequences. Subsequently, specific nucleic acid probes targeting EBER were applied for hybridization. After hybridization, stringent washing steps were performed to remove nonspecifically bound probes, and signal detection was conducted using an enzyme-labeled antibody system. Results were evaluated under a microscope. Clear and specific staining in tumor cells was interpreted as EBER-positive (EBER+), whereas the absence of specific staining in tumor cells was defined as EBER-negative (EBER−). Positive and negative controls were included in each run to ensure the accuracy and reproducibility of the assay.

### 10x visium CytAssist spatial transcriptomics and spatial metabolomics sample preparation

Freshly collected NPC and CIH tissue blocks were embedded in Cryo-Gel (Leica, Germany) and stored at -80 °C until sectioning. Sections of approximately 10 μm thickness were prepared using a cryostat microtome (Leica CM 1950, Leica Microsystems, Germany), mounted on positively charged desorption plates, and stored at -80 °C for subsequent analysis.

### 10x visium CytAssist spatial transcriptomics sequencing

The tissue sections were methanol-fixed, H&E-stained, scanned, and decolorized following the 10x Genomics protocol. Using the Visium CytAssist Spatial Gene Expression reagent kit, probes were hybridized, released, and transferred to Visium CytAssist slides for library construction. DNA libraries were sequenced using the PE-150 mode for high-throughput sequencing.

### Spatial mass spectrometry imaging

The MSI analysis was conducted with reference to the study by Luo et al. (14). The experiment was performed using a Waters-DESI platform (Beijing Victor Technology Co., Ltd., Beijing, China) coupled with a SYNAPT XS mass spectrometer (Q Exactive, Thermo Scientific, U.S.A). In negative ion mode, the spray solvent was acetonitrile (ACN)/H_2_O (8:2), and in positive ion mode, the spray solvent was ACN/H_2_O (8:2). The solvent flow rate was 2 μL/min, and the spray gas pressure was set to 0.6 MPa. The distance from the sprayer to the sample surface was 3 mm, and the distance from the sprayer to the ion transfer tube was 5 mm. The MS resolution was set to 20, 000, with a mass range of 70–1200 Da. The ionization source temperature was 150°C, and the tube temperature was 40°C. During MSI scanning, the spatial resolution (μm*μm) was 100*100.

### Mass spectrometry data processing

The raw mass spectrometry data files in “.raw” format were converted to “.imzML” format using imzMLConverter and then imported into MSiReader software (15). After background subtraction using the Cardinal package, ion images were reconstructed (16). QC samples were prepared from homogenized tissue of the corresponding experimental samples. A QC spot was placed adjacent to each experimental sample to enable batch correction. The FindIntegrationAnchors function in the Seurat package was used to identify anchors between the experimental and QC samples, and the IntegrateData function was subsequently applied to integrate the datasets based on these anchors, thereby constructing an integrated model to align data across different batches. Region-specific MS profiles were precisely extracted by matching high-spatial-resolution H&E images.

### Metabolite annotation

High-resolution mass spectrometry imaging data were annotated using the LuMet-Space 1.0 database and the pySM annotation framework in combination with mass2adduct (https://github.com/kbseah/mass2adduct) (17). The mass deviation between the measured and theoretical m/z values was controlled within 10 ppm. The pySM framework was employed to identify and annotate adduct ions of metabolites from the mass spectrometry data. The final adduct forms were selected based on similarity scores, with corresponding scoring metrics including moc, spec, spat, and msm parameters. The LuMet-Space 1.0 database is a dedicated spatial metabolomics database covering different tissue types from humans, animals, and plants, established through literature curation, manual verification, and systematic literature retrieval.

### Mass spectrometry imaging acquisition and scanning

Mass spectrometry imaging (MSI) data were acquired using the platform control software in a line-by-line scanning mode. Based on the predetermined dimensions of the tissue section, the X- and Y-axis scan lengths were defined, and data were collected sequentially for each line. After scanning a line, the sample stage rapidly returned to the starting position at a speed of 10 mm/s to prevent contamination or damage to unscanned areas. The stage then stepped along the Y-axis according to the specified spatial resolution, and this process was repeated until the entire tissue section was scanned, ensuring accurate and reliable MSI results. Data acquisition was performed using MassLynx software and processed with the High Definition Imaging (HDI) system. The acquisition sequence was configured according to sample dimensions, step size, and scan speed, with detailed DESI ion source and mass spectrometry acquisition parameters for both positive and negative ion modes provided in the corresponding table.

### Spatial shrunken centroids clustering

Spatial metabolomics data, typically organized as a high-dimensional M × N matrix (M molecular features × N spatial pixels), were analyzed using Spatial Shrunken Centroids Clustering (SSCC) with the Cardinal package (v2.14.0). SSCC integrates pixel-level molecular expression profiles with spatial neighborhood information by calculating distances in the high-dimensional feature space and incorporating a spatial shrinkage parameter to enforce spatial continuity. Spatially adjacent pixels with similar molecular characteristics are then clustered, enabling automatic segmentation of tissue regions. This approach effectively identifies spatially coherent functional regions and provides a robust foundation for subsequent region selection, feature extraction, and biological interpretation.

### Differential metabolite analysis

Differential metabolites between groups were identified using orthogonal partial least squares discriminant analysis (OPLS-DA) with the Ropls package (v1.30.0). Variable Importance of Projection (VIP) values from the OPLS-DA model were used to assess the contribution of each metabolite to group separation. Metabolites with VIP > 1 and P < 0.05 were further validated using two-tailed Student’s T-tests.

### 10x visium CytAssist spatial transcriptomics gene quantification

Spatial transcriptomics data were processed using Space Ranger (v2.0.1), which aligned reads to the reference genome (human: GRCh38) and distinguished spatial barcodes for each spot. Quality metrics, including total spot count, pair reads per spot, detected genes, and UMI counts, were evaluated to ensure data quality.

### Single-cell and spatial cell type annotation

Based on Space Ranger’s initial quality control, further processing was conducted using the Seurat package (18). Data normalization was performed with the SCTransform function (19), followed by PCA using the top 3000 highly variable genes (HVGs). Batch correction was applied using the RunHarmony function, and clustering was subsequently performed.

Single-cell transcriptome data from 9 samples (7 NPC and 2 CIH) were filtered, quality-controlled, and batch-corrected using Harmony, resulting in 89, 875 cells for clustering and annotation with Scanpy. Spatial cell type deconvolution was performed using RCTD (20), adjusting for differences in sequencing technologies.

### Single-cell transcriptome data analysis

Scores for Proliferation, Adipogenesis, Angiogenesis, Apoptosis, Fatty Acid, Glycolysis, Hypoxia, and Oxidative Phosphorylation were calculated using the AddModuleScore function. RNA velocity analysis was conducted with velocyto and scvelo using default parameters, mapping results onto UMAP plots for consistency in visualization. Transcription factor networks were constructed from single-cell expression matrices using pySCENIC, while scFEA was employed to infer cellular metabolic flux and metabolite abundance. CellPhoneDB was utilized to infer cell-cell interactions, and ktplots were used for visualization. Highly expressed genes in malignant epithelial cells were calculated using FindAllMarkers, with parameters set to logfc.threshold=0.25 and test.use=‘wilcox’.

### Glycerophospholipid metabolism-related gene screening

Differential gene expression analysis between NPC and CIH was performed using the FindMarkers function in Seurat. Genes with P < 0.05 and fold change (FC) > 1.5 were considered significantly differentially expressed in the NPC vs. CIH comparison. Similarly, differential gene expression analysis between EBER+ and EBER− groups was conducted using the same method. Genes with P < 0.05 and FC > 1.5 were defined as significantly differentially expressed in the EBER+ vs. EBER- comparison.

Gene set REACTOME_GLYCEROPHOSPHOLIPID_BIO-SYNTHESIS.v2023.2.Hs.gmt was downloaded from the GSEA database, comprising 128 glycerophospholipid-related genes. These genes were intersected with the upregulated genes identified in NPC compared with CIH, as well as with the upregulated genes in EBER+ versus EBER- samples. The resulting two intersecting gene sets were combined by taking their union for subsequent analyses.

The union gene set was further filtered according to the following criteria: (1) genes highly expressed in tumor cell-enriched regions based on DSP spatial transcriptomics data; (2) genes significantly upregulated in NPC tissues compared with normal tissues in the GSE12452 dataset; and (3) genes whose higher expression levels were associated with poorer overall survival in NPC patients, as determined using DSP spatial transcriptomics data.

Survival analysis was performed using the survival and survminer R packages. Gene expression differences were visualized using box plots generated with ggplot2 and ggpubr, and ROC curves were constructed using the pROC package.

### Spatial pseudotime analysis

Spatial trajectory inference was performed using the stLearn framework. After SME normalization and spatial clustering, partition-based graph abstraction (PAGA) was applied to model the global topology of spatial relationships across tissue sections (21, 22). To infer trajectory directionality, diffusion pseudotime (DPT) was calculated. The root of the spatial trajectory was defined based on both histopathological annotation and spatial cell composition. Specifically, spots located in tumor-immune mixed regions were selected as the starting point, as these regions represent transitional niches between malignant epithelial cells and immune-enriched regions. This selection was supported by hematoxylin and eosin (H&E) staining as well as RCTD-based cell type deconvolution, which demonstrated the co-localization of malignant epithelial cells and immune cell populations within these regions. To further incorporate spatial context, pseudo-space-time distance (PSTD) was computed by integrating transcriptomic similarity with physical spatial distance (23). Directed trajectories were reconstructed using a minimum spanning tree algorithm to identify the shortest rooted tree and branching structures. To evaluate the robustness and biological relevance of the inferred trajectories, we analyzed gene expression dynamics along pseudotime and performed functional enrichment analyses on branch-associated genes.

## Results

### Single-cell spatial transcriptomic and metabolic profiles in the microenvironment of NPC and CIH

**Figure 1:**
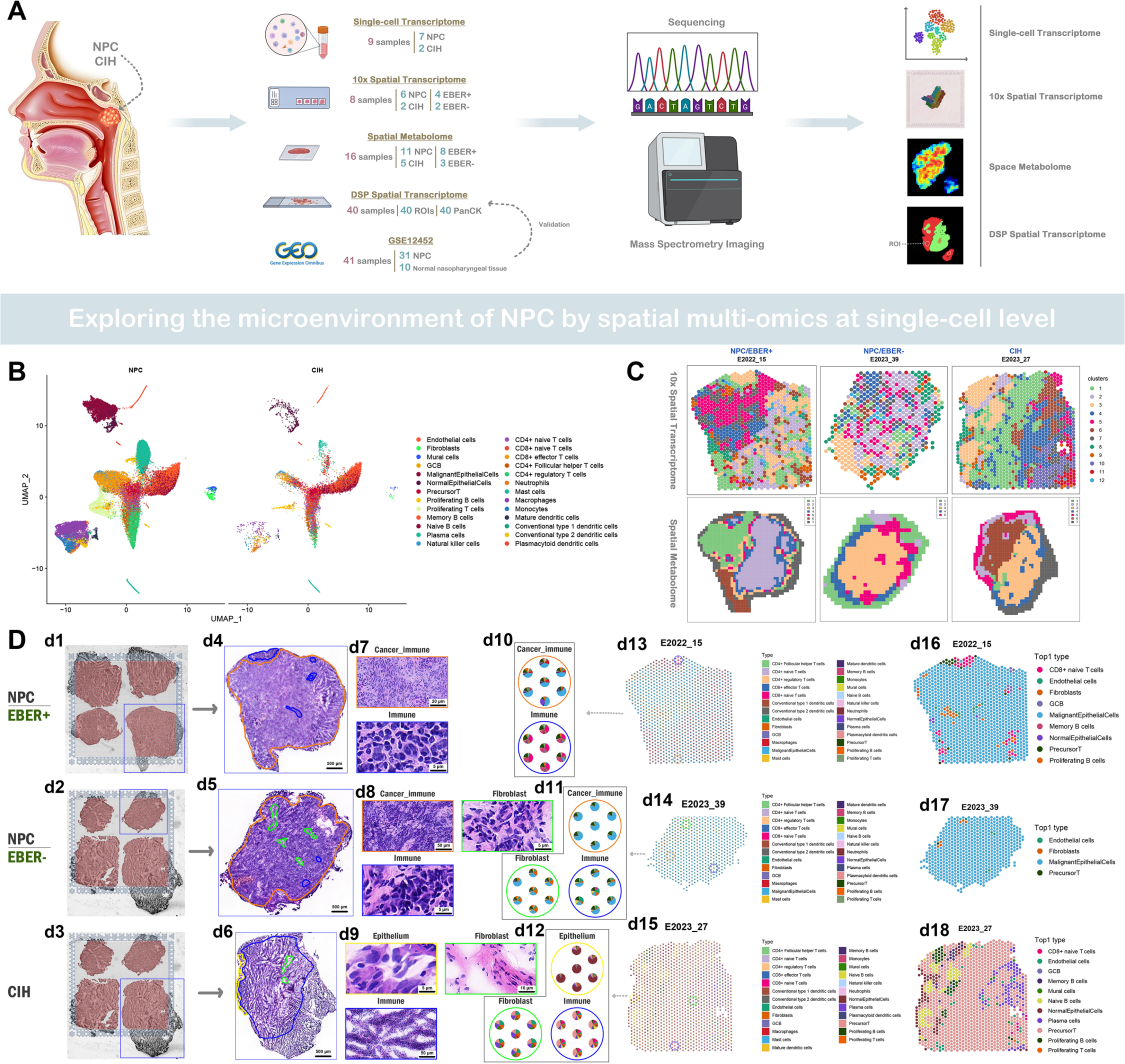
Single-cell spatial transcriptomic and metabolic profiles in the microenvironment of NPC and CIH. **(A)** A flowchart depicting the research methodology. **(B)** Single-cell transcriptomic analysis: Cell atlas for NPC (left) and CIH (right), where each dot represents an individual cell, and colors denote distinct cell populations. **(C)** 10x spatial transcriptomics clustering map (top): Each dot represents a spatial spot, colored by cluster distinction. Spatial metabolomics clustering map (bottom): Each square represents a pixel, with colors indicating different clusters. **(D)** Images d1-d3 display the tissue regions captured on the chip (10x spatial transcriptomics). Images d4-d9 present hematoxylin and eosin (HE) staining, where orange indicates tumor-immune regions, blue signifies immune regions, green represents fibroblast regions, and yellow depicts epithelial regions. Images d10-d15 consist of pie charts illustrating the cell type composition at each spatial slice, with colors corresponding to HE-stained regions. Images d16-d18 highlight the predominant cell type (Top1) in each spot, with colors representing different cell types.

We analyzed samples from nine untreated patients, comprising seven nasopharyngeal carcinoma (NPC) tissues and two cases of nasopharyngeal chronic inflammation with hyperplasia (CIH), using single-cell transcriptome sequencing (**Supplementary Table 1**). Additionally, we collected eight untreated samples for 10x spatial transcriptome sequencing, which included four EBER+ and two EBER- samples. Spatial metabolomics studies were conducted on 16 untreated samples (11 NPC and 5 CIH), consisting of eight EBER+ and three EBER- samples (**Supplementary Table 2**). To supplement our analysis, we utilized previously acquired digital spatial transcriptomics data for prognostic predictions of target genes (**Figure 1A**) (13). Following data quality control and filtering, we performed dimensionality reduction, clustering, and annotation on 89, 875 cells, which were categorized into 26 distinct cell clusters: Endothelial cells, Fibroblasts, Mural cells, Germinal Center B cells (GCB), Malignant Epithelial Cells, Normal Epithelial Cells, Precursor T cells, Proliferating B cells, Proliferating T cells, Memory B cells, Naive B cells, Plasma cells, Natural Killer cells, CD4+ naive T cells, CD8+ naive T cells, CD8+ effector T cells, CD4+ Follicular helper T cells, CD4+ regulatory T cells, Neutrophils, Mast cells, Macrophages, Monocytes, Mature dendritic cells, Conventional type 1 dendritic cells, Conventional type 2 dendritic cells, and Plasmacytoid dendritic cells (**Figure 1B**). The 10x spatial transcriptome data were similarly clustered into 12 groups, each displaying consistent gene expression patterns. The SSCC clustering of spatial metabolomics data identified 5–7 distinct clusters, with each cluster demonstrating similar metabolite abundances (**Figure 1C**). Hematoxylin and eosin (H&E) staining of tissue samples from NPC/EBER+, NPC/EBER-, and CIH patients allowed pathologists to categorize NPC tissues into immune cell-enriched regions, mixed tumor and immune cell regions, and fibroblast-enriched regions. CIH tissues were classified into epithelial cell-enriched, immune cell-enriched, and fibroblast-enriched regions (**Figure 1D** d1-d9). Using RCTD, we mapped cell cluster information obtained from single-cell data onto spatial transcriptome slices, estimating the proportion of each cell type at each spatial location. The distribution of cell clusters on the slices corresponded with regions identified in H&E staining (**Figure 1D** d10-d15). The Top1 cell type represents the most abundant cell type in each spot, while the Top2 cell type is the second most prevalent. If the Top1 cell type comprises more than 70% of any spot, it replaces the Top2 cell type. In NPC tissues, the predominant cell type was typically malignant epithelial cells, whereas in CIH tissues, precursor T cells were more prevalent. Notably, EBER+ NPC tissues exhibited greater diversity and abundance of immune cells compared to EBER- NPC tissues (**Figure 1D** d16-d18; **Supplementary Figure S1A**).

### Cellular composition and characterization in the NPC tumor microenvironment

**Figure 2:**
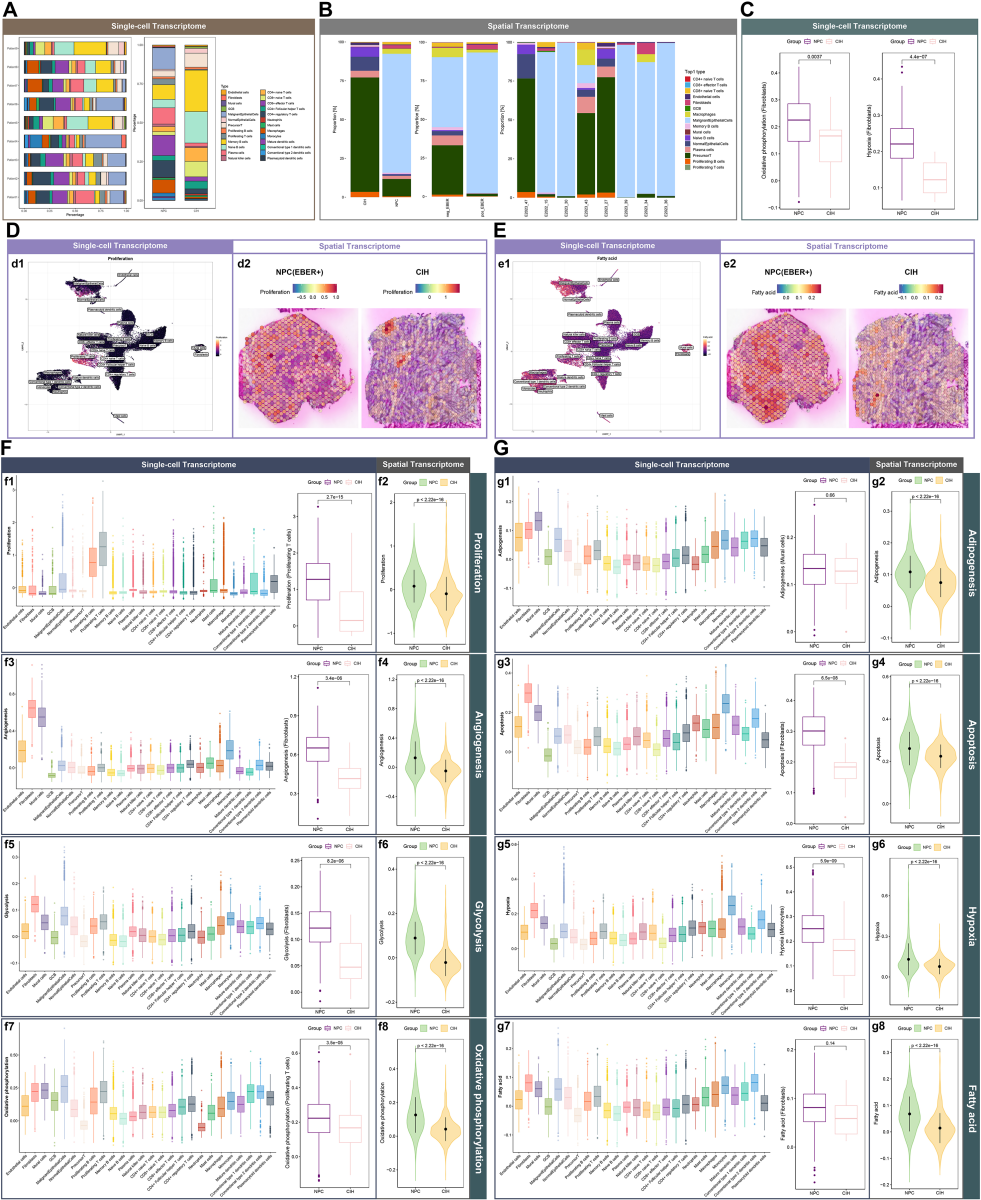
Cellular composition and characterization within the NPC tumor microenvironment. **(A)** Single-cell transcriptomic analysis: Proportions of cells from 9 samples (left) and various groups (right), with different colors indicating distinct cell clusters. **(B)** Spatial transcriptomic analysis: Proportions of Top1 cells across different groups (left and center) and in 8 samples (right), using distinct colors for different Top1 cell types. **(C)** Comparison of oxidative phosphorylation and hypoxia scores in fibroblasts between NPC and CIH, analyzed using the Wilcoxon rank-sum test. **(D)** UMAP plot of proliferation scores across 26 cell clusters, where each point represents a cell, and a yellow hue indicates higher scores (d1). Proliferation scores for two tissue sections, represented by spots, with red indicating higher scores (d2). **(E)** UMAP plot of fatty acid scores across 26 cell clusters, where each point represents a cell, with yellow indicating higher scores (e1). Fatty acid scores for two tissue sections, with spots indicating higher scores in red (e2). **(F, G)** Single-cell transcriptomic analysis: Boxplots of proliferation, adipogenesis, angiogenesis, apoptosis, fatty acid, glycolysis, hypoxia, and oxidative phosphorylation scores for 26 cell clusters (left: f1, f3, f5, f7, g1, g3, g5, g7). Boxplots illustrating intergroup differences in proliferation and oxidative phosphorylation scores for proliferating T cells, assessed using the Wilcoxon rank-sum test (right: f1 and f7). Boxplots displaying intergroup differences in angiogenesis, glycolysis, apoptosis, and fatty acid scores for fibroblasts (right: f3, f5, g3, and g7). Boxplot illustrating intergroup differences in adipogenesis scores for mural cells (right: g1). Boxplot showing intergroup differences in hypoxia scores for monocytes (right: g5). Spatial transcriptomic analysis: Violin plots showing intergroup differences in scores for various processes, analyzed using the Wilcoxon rank-sum test (f2, f4, f6, f8, g2, g4, g6, and g8).

In the single-cell transcriptome analysis, the proportions of Malignant Epithelial Cells, Proliferating T cells, Plasma cells, CD8+ effector T cells, CD4+ regulatory T cells, and Macrophages were higher in NPC compared to CIH. Conversely, the proportions of Normal Epithelial Cells, Precursor T cells, Memory B cells, Naive B cells, CD4+ naive T cells, CD8+ naive T cells, and CD4+ Follicular helper T cells were higher in CIH than in NPC. This cellular composition exhibited heterogeneity across different patients. The proportion of Fibroblasts in the microenvironment was low in both NPC and CIH; however, it was higher in NPC tissues (**Figure 2A**). In the spatial transcriptome analysis, the top cell types of Fibroblasts, Macrophages, and Malignant Epithelial Cells were more prevalent in NPC compared to CIH. The proportions of Fibroblasts and Malignant Epithelial Cells as top cell types were greater in EBER+ NPC than in EBER- NPC. Additionally, the top cell types displayed variability among patients (**Figure 2B**). A relationship between Fibroblasts and Malignant Epithelial Cells was observed in the NPC microenvironment. We calculated tumor feature scores, including Proliferation, Adipogenesis, Angiogenesis, Apoptosis, Fatty Acid metabolism, Glycolysis, Hypoxia, and Oxidative Phosphorylation. In the spatial transcriptome, these scores were generally higher in NPC compared to CIH (**Figures 2F** f2, f4, f6, f8, **2G** g2, g4, g6, g8). From the single-cell transcriptome, we selected cell clusters with the highest scores for these features, including Proliferating T cells, Fibroblasts, Mural cells, Malignant Epithelial Cells, and Monocytes. Malignant Epithelial Cells had the highest score for Oxidative Phosphorylation; however, due to their absence in CIH, we selected Proliferating T cells for further analysis (**Figure 2F** f1, f3, f5, f7 and **Figure 2G** g1, g3, g5, g7). Proliferating T cells in NPC exhibited higher Proliferation and Oxidative Phosphorylation scores than those in CIH (**Figures 2F** f1, f7, **2D**). The Hypoxia score of Monocytes in NPC was also elevated compared to CIH (**Figure 2G** g5). Fibroblasts demonstrated the highest scores for Angiogenesis, Glycolysis, Apoptosis, and Fatty Acid metabolism, with scores for Angiogenesis, Glycolysis, Apoptosis, Oxidative Phosphorylation, and Hypoxia being higher in NPC than in CIH. Notably, the Fatty Acid score for Fibroblasts in NPC was higher than in CIH, but did not reach statistical significance (p < 0.05) (**Figures 2C, E, F** f3, f5, **2G** g3, g7). The microenvironment of NPC is highly complex, characterized by diverse interactions among different cell types. Malignant cell proliferation may increase metabolic demand and competition for nutrients within the tumor microenvironment. In the early stages, CD8+ T cells proliferate and exert cytotoxic effects, but they may eventually become exhausted (7, 24). Fibroblasts showed relatively higher glycolipid metabolism-related scores in the NPC microenvironment, which may be associated with hypoxia, apoptosis, and angiogenesis.

### The accumulation of fatty acids in the NPC microenvironment is associated with fibroblasts expressing high levels of CCL11

**Figure 3:**
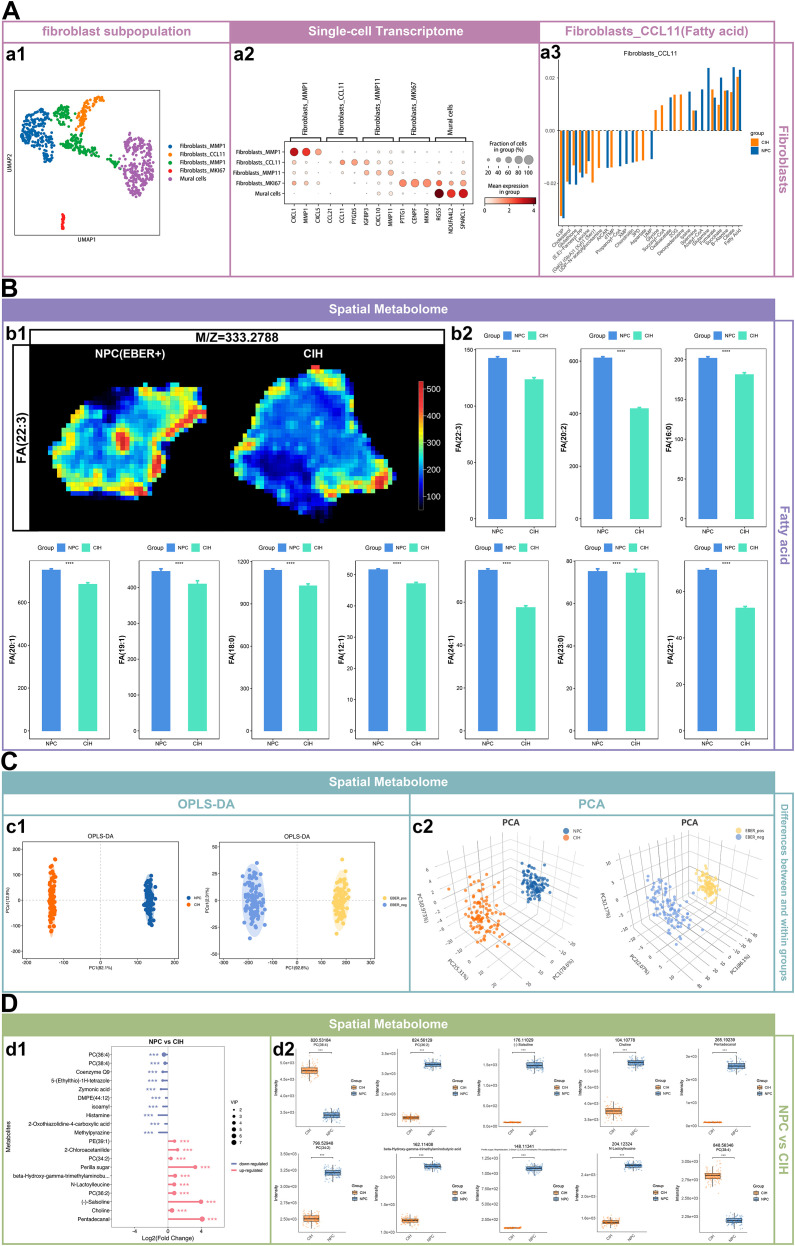
Accumulation of fatty acids in the NPC microenvironment associated with CCL11-expressing fibroblasts. **(A)** UMAP plot of fibroblasts and mural cells, with each point representing a cell and colors denoting different cell clusters (a1). The top three differentially expressed genes for each cell cluster are shown (a2). A bar plot displays the average abundance of the top 10 accumulated and depleted metabolites in Fibroblasts_CCL11 across groups (a3). **(B)** Imaging of fatty acid (FA 22:3) in NPC (EBER+) and CIH, with colors from dark blue to dark red indicating increasing metabolite abundance (b1). Differences in fatty acid types between groups, analyzed using the Wilcoxon rank-sum test, where “ns” indicates p > 0.05, “*” indicates p ≤ 0.05, “**” indicates p ≤ 0.01, “***” indicates p ≤ 0.001, and “****” indicates p ≤ 0.0001 (b2). **(C)** Orthogonal partial least squares discriminant analysis (OPLS-DA): PC1 reflects intergroup differences, while PCo1 captures intra-group variation, with colors representing different groups (c1). Principal component analysis (PCA): PC1 represents the variance explained by the first principal component, PC2 by the second, and PC3 by the third, with colors indicating different groups (c2). **(D)** Top 10 upregulated and downregulated metabolites with the highest variable importance in projection (VIP) scores in NPC vs. CIH, with dot size reflecting VIP value (d1). Box plots of the top 10 significantly different metabolites ranked by VIP in NPC vs. CIH (d2).

In our analysis, we identified 270 mural cells and 516 fibroblasts, which were categorized into four distinct clusters: Fibroblasts_MMP1, Fibroblasts_CCL11, Fibroblasts_MMP11, and Fibroblasts_MKI67, based on the top three differentially expressed genes in each cluster (**Figure 3A** a1 and a2). According to scFEA-based predictions of metabolic flux within these fibroblast clusters, fatty acids were predicted to be among the most enriched metabolites in Fibroblasts_CCL11, with a trend toward higher levels in NPC samples (**Figure 3A** a3). Similarly, Fibroblasts_MMP11 and Fibroblasts_MMP1 also showed fatty acid as their second and fourth most abundant metabolites, respectively, while Fibroblasts_MKI67 ranked fatty acid as the third (**Supplementary Figure S1B**). The spatial metabolomics analysis revealed that several fatty acids, including FA (22:3), FA (20:2), FA (16:0), FA (20:1), FA (19:1), FA (18:0), FA (12:1), FA (24:1), FA (23:0), and FA (22:1), were significantly more abundant in NPC compared to CIH (**Figure 3B**). Results from Orthogonal Partial Least Squares Discriminant Analysis (OPLS-DA) and Principal Component Analysis (PCA) indicated significant differences in metabolite profiles between NPC and CIH groups, as well as between EBER+ and EBER- NPC groups (**Figure 3C**). Differential metabolites were identified based on the criteria of P < 0.05, VIP > 1, and fold change (FC) = 1.2. Notably, many common differential metabolites were found between the two comparison groups (NPC vs. CIH and EBER+ NPC vs. EBER- NPC). Among the top 10 differential metabolites, four key metabolites—PC(36:4), beta-Hydroxy-gamma-trimethylaminobutyric acid, PC(38:4), and choline—exhibited significant differences in both comparison groups (**Figure 3D**; **Supplementary Figure S1C**).

### Fibroblast developmental trajectory, spatial localization, and communication network

**Figure 4:**
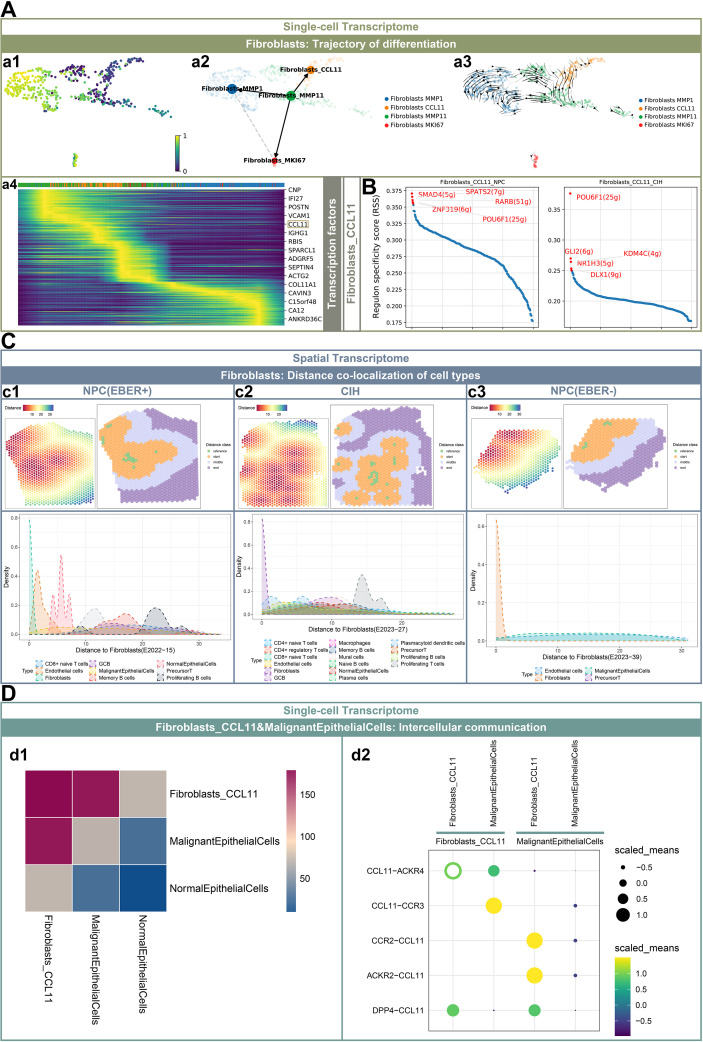
Fibroblast developmental trajectory, spatial localization, and communication network. **(A)** Visualization of fibroblast developmental trajectory from early (dark purple) to late (yellow), with each dot representing a cell (a1). Differentiation trajectories of fibroblast clusters, with arrows indicating the direction of differentiation and colors representing different clusters (a2 and a3). A heatmap of genes exhibiting significant shear kinetics over time is presented (a4). **(B)** The top five regulons of Fibroblasts_CCL11 in NPC and CIH. **(C)** Euclidean distances of fibroblasts from surrounding cells in EBER+ NPC (c1), CIH (c2), and EBER- NPC (c3) tissues, represented from red (near) to blue (far) (c1-c3: top left). Using fibroblasts as a reference, distances are categorized into four tiers from 0 (reference) to farthest (c1-c3: top right). Density plots of cell types at varying distances from fibroblasts, color-coded by cell type (c1-c3: bottom). **(D)** The number of ligand-receptor pairs interacting between Fibroblasts_CCL11, Malignant Epithelial Cells, and Normal Epithelial Cells (d1). The ligand-receptor pairs containing CCL11 that interact between Fibroblasts_CCL11 and Malignant Epithelial Cells are highlighted (d2).

Differentiation trajectories inferred from RNA velocity analysis suggested a transition from Fibroblasts_MMP11 toward Fibroblasts_MMP1, Fibroblasts_CCL11, and Fibroblasts_MKI67, with Fibroblasts_MMP1 representing a potential terminal state. During this pseudotime progression, the expression level of CCL11 increased initially and then decreased (**Figure 4A**). The top five transcription factors associated with Fibroblasts_CCL11 in NPC were identified as SMAD4, SPATS2, RARB, ZNF319, and POU6F1, with POU6F1 also being prominent in CIH (**Figure 4B**). Active transcription factors in Fibroblasts_MMP11, Fibroblasts_MMP1, and Fibroblasts_MKI67 are detailed in **Supplementary Figure 2A**. To explore cellular interactions, we calculated the Euclidean distances between Fibroblasts or Malignant Epithelial Cells and surrounding cell types, categorizing distances into 2–4 levels from near to far to infer co-localization patterns (**Figure 4C**, **Supplementary Figure S2B**). In EBER+ NPC, Fibroblasts displayed decreased density with increasing distance, with endothelial cells and malignant epithelial cells being in closer proximity (**Figure 4C** c1). In contrast, CIH exhibited a greater diversity of cell types surrounding Fibroblasts, including plasmacytoid dendritic cells, endothelial cells, and proliferating B cells (**Figure 4C** c2). For EBER- NPC, the proximity of endothelial cells and malignant epithelial cells to Fibroblasts was also noted (**Figure 4C** c3). When using Malignant Epithelial Cells as a reference in EBER+ NPC, we observed that their density diminished with increasing distance, while B cell clusters (including memory B cells, germinal center B cells, and proliferating B cells) were located nearer to Malignant Epithelial Cells (**Supplementary Figure S2B** b1). In EBER- NPC, the density of Malignant Epithelial Cells decreased with distance, while fibroblast and endothelial cell densities increased (**Supplementary Figure S2B** b2). Using CellPhoneDB to infer interactions between Fibroblasts_CCL11 and Malignant Epithelial Cells, we observed a higher number of predicted interactions with Malignant Epithelial Cells compared to Normal Epithelial Cells (**Figure 4D** d1). In the NPC microenvironment, predicted interactions involving Fibroblasts_CCL11 included CCL11-ACKR4 and DPP4-CCL11 ligand-receptor pairs. The interactions between Fibroblasts_CCL11 and Malignant Epithelial Cells were predicted to involve CCL11-ACKR4 and CCL11-CCR3 interactions, while Malignant Epithelial Cells were predicted to interact with Fibroblasts_CCL11 via CCR2-CCL11, ACKR2-CCL11, and DPP4-CCL11 ligand-receptor pairs (**Figure 4D** d2).

### Glycerophospholipid-related genes are implicated in malignant epithelial cells in NPC

**Figure 5:**
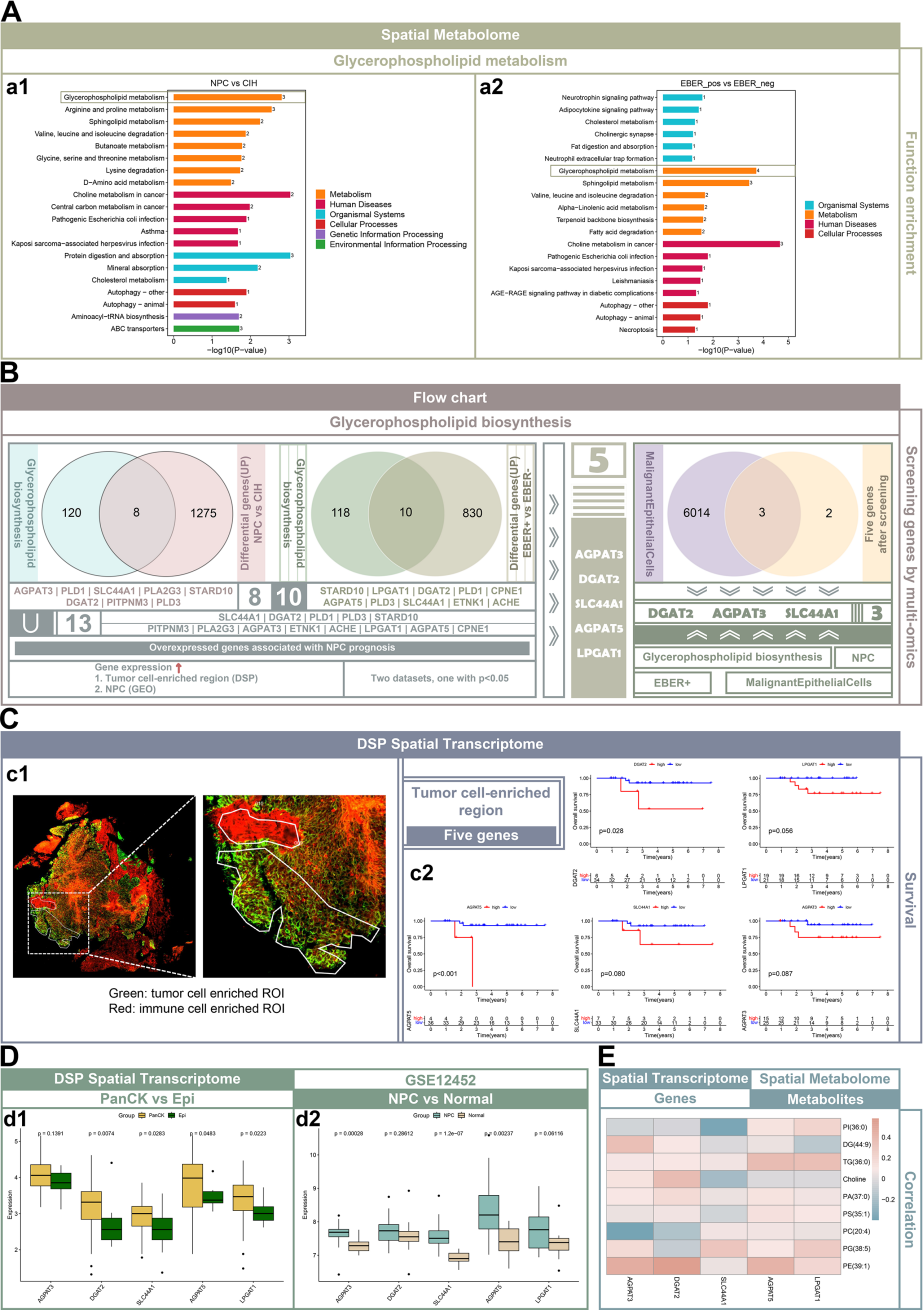
Role of glycerophospholipid-related genes in malignant epithelial cells of EBER+ NPC. **(A)** KEGG enrichment analysis of differential metabolites in NPC vs. CIH, displaying the top 20 pathways (a1). KEGG functional enrichment of differential metabolites in EBER+ NPC vs. EBER- NPC, showing the top 20 pathways (a2). **(B)** Flowchart outlining the screening process for glycerophospholipid-related genes. **(C)** Regions of interest (ROIs) identified in DSP spatial transcriptomics (c1). Kaplan-Meier survival analysis in NPC patients based on glycerophospholipid-related gene expression (log-rank test) (c2). **(D)** Box plots validating the expression of five glycerophospholipid-related genes in DSP spatial transcriptomics, comparing tumor-enriched and normal epithelial regions (Wilcoxon rank-sum test) (d1). Validation of these genes in the GEO dataset (GSE12452), comparing NPC and normal nasopharyngeal tissue (d2). **(E)** Pearson correlation analysis between glycerophospholipid-related genes and metabolites in NPC, with correlations color-coded from negative (gray-blue) to positive (gray-red).

KEGG pathway enrichment analysis demonstrated that the differential metabolites identified in both the EBER+ NPC versus EBER- NPC and NPC versus CIH groups were significantly enriched in glycerophospholipid metabolism pathways (**Figure 5A** a1 and a2). From the 10x spatial transcriptomics data, we identified 1, 283 genes significantly upregulated in NPC (P < 0.05, fold change [FC] > 1.5) and 840 genes significantly upregulated in the EBER+ group (P < 0.05, FC > 1.5) (**Supplementary Figure S2C**). We then retrieved the “REACTOME_GLYCEROPHOSPHOLIPID_BIOSYNTHESIS.v2023.2.Hs.gmt” file from the GSEA database, which includes 128 glycerophospholipid-related genes. By intersecting these genes with the 1, 283 NPC-upregulated genes, we identified 8 overlapping genes. Similarly, intersecting the 128 glycerophospholipid-related genes with the 840 EBER+ upregulated genes yielded 10 overlapping genes. This process resulted in a total of 13 glycerophospholipid-related genes.

To refine our selection further, we applied the following criteria to identify 5 glycerophospholipid-related genes: genes for which higher expression in tumor cell-enriched regions correlates with poorer prognosis in NPC patients and those that are either more highly expressed in tumor cell-enriched regions compared to normal epithelial regions (as indicated by DSP spatial transcriptomics) or more highly expressed in NPC compared to normal nasopharyngeal tissue (from the GSE12452 dataset). The identified genes were AGPAT3, DGAT2, SLC44A1, AGPAT5, and LPGAT1 (**Figure 5B**). These genes exhibited consistently high expression levels in NPC samples (10x spatial transcriptomics), in tumor cell-enriched regions (DSP spatial transcriptomics), and their elevated expression was associated with poorer prognosis in NPC patients. Moreover, they demonstrated higher expression in NPC samples compared to normal tissues (GSE12452). Although some P-values exceeded 0.05 due to sample size limitations, the expression trends remained consistent (**Figures 5C, D**, **Supplementary Figure S2C**). In the multivariate Cox regression analysis, the five-gene signature (riskScore) showed a trend toward worse prognosis (HR = 1.374, 95% CI: 0.916-2.061), though it did not reach statistical significance (P = 0.125), potentially due to limited sample size (**Supplementary Figure S3B**).

In the single-cell transcriptome analysis, Fibroblasts_CCL11 were predicted to exhibit ligand-receptor interactions with Malignant Epithelial Cells. By intersecting the highly expressed genes in Malignant Epithelial Cells with the previously identified five genes, we pinpointed AGPAT3, DGAT2, and SLC44A1. These three genes exhibited high expression levels in Malignant Epithelial Cells of EBER+ NPC, with their elevated expression in tumor-enriched regions correlating with poorer prognosis for NPC patients (**Figure 5C**). Further correlation analysis showed that AGPAT3 expression was positively associated with PE(39:1) and DG(44:9), DGAT2 with PE(39:1) and choline, SLC44A1 with PG(38:5), AGPAT5 with PE(39:1) and TG(36:0), and LPGAT1 with TG(36:0) and PG(38:5) (**Figure 5E**).

### Pathway regulation of glycerophospholipid-related genes and associated metabolites

**Figure 6:**
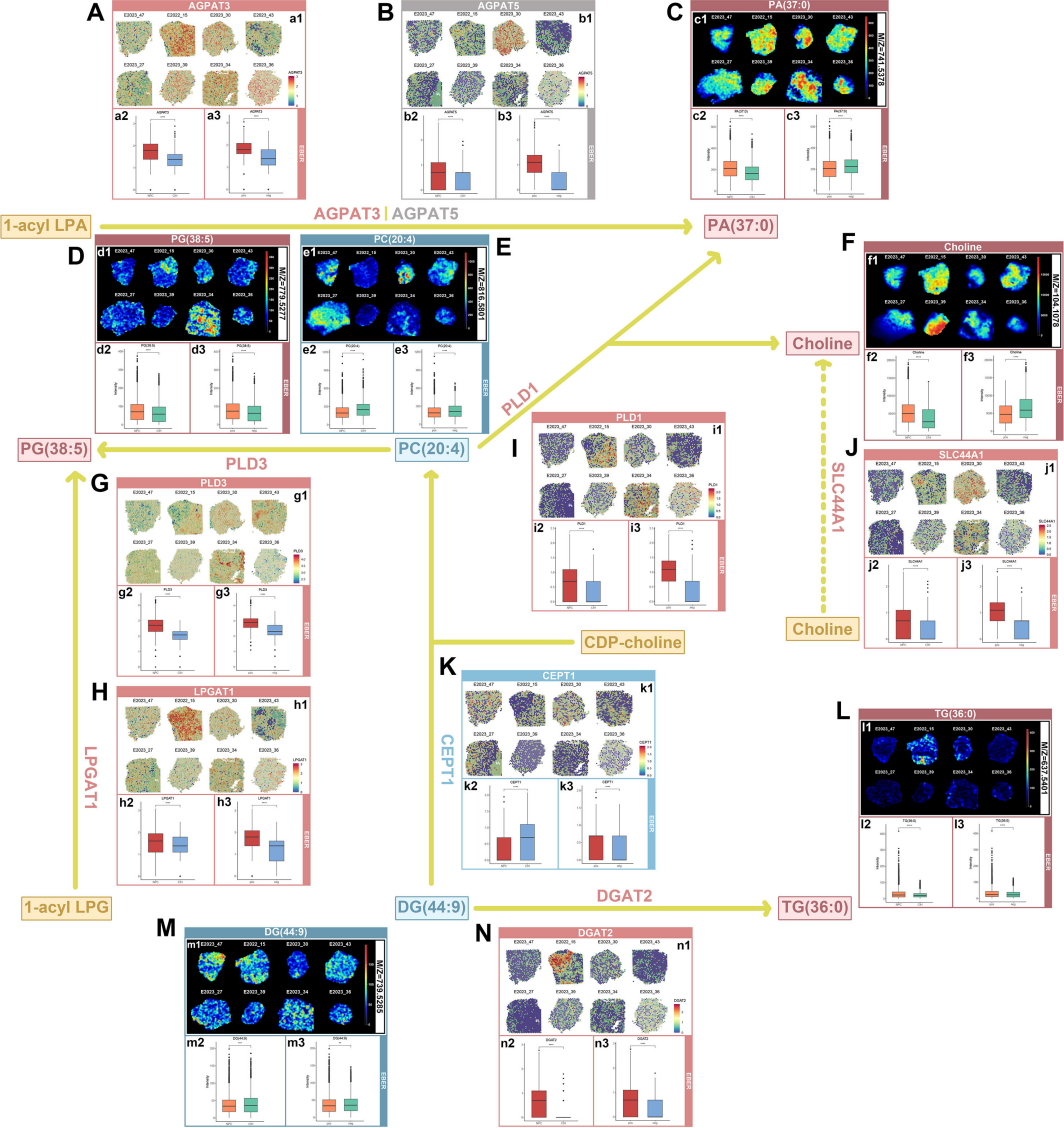
Pathways of glycerophospholipid-related genes and metabolites. **(A–N)** This figure illustrates the glycerophospholipid biosynthesis pathway, featuring spatial expression maps of glycerophospholipid-related genes (a1, b1, g1, h1, i1, j1, k1, and n1) and spatial abundance maps of their associated metabolites (c1, d1, e1, f1, l1, and m1). The spatial expression differences of glycerophospholipid-related genes between NPC samples (EBER+ and EBER-) and CIH were analyzed using spatial transcriptomics data, with statistical significance assessed via the Wilcoxon rank-sum test (a2, a3, b2, b3, g2, g3, h2, h3, i2, i3, j2, j3, k2, k3, n2, and n3). Additionally, the differences in spatial abundance of glycerophospholipid-related metabolites between NPC and CIH were evaluated using spatial metabolomics data, with significance determined by the Wilcoxon rank-sum test (c2, c3, d2, d3, e2, e3, f2, f3, l2, l3, m2, and m3).

We inferred a putative pathway involving five glyceropho-spholipid-related genes and their corresponding metabolites based on integrated transcriptomic and metabolomic data. AGPAT3 and AGPAT5 are known to catalyze the conversion of 1-acyl LPA to phosphatidic acid (PA(37:0)). In our dataset, AGPAT3 is upregulated in NPC samples (**Figure 6A**), whereas AGPAT5 is specifically upregulated in EBER+ NPC samples (**Figure 6B**). Consistently, PA(37:0) is also elevated in NPC (**Figure 6C**), suggesting a potential association between gene expression and metabolite abundance. Additionally, NPC tissues displayed increased PG(38:5) levels (**Figure 6D**), decreased PC(20:4) abundance (**Figure 6E**), and elevated choline contents (**Figure 6F**).

PLD3 expression was significantly increased in NPC (**Figure 6G**). Mechanistically, PLD3 mediates the catabolism of PC(20:4) to generate PG(38:5). Similarly, LPGAT1 was upregulated in NPC (**Figure 6H**); LPGAT1 catalyzes the transformation of 1-acyl lysophosphatidic acid (LPG) into PG(38:5), which corroborates the increased PG(38:5) levels in NPC (**Figure 6D**). PLD1 was also overexpressed in NPC specimens (**Figure 6I**). PLD1-driven hydrolysis of PC(20:4) produces PA(37:0) and choline, which is consistent with the upregulated abundance of PA(37:0) and choline in NPC (**Figures 6C, F**). Moreover, the choline transporter gene SLC44A1 was upregulated in NPC (**Figure 6J**), which accounts for the enhanced intracellular choline availability in tumor cells.

In contrast, CEPT1 expression was downregulated in NPC (**Figure 6K**). CEPT1 is responsible for synthesizing PC(20:4) from DG(44:9) and CDP-choline, and its downregulation explains the decreased PC(20:4) content in NPC (**Figure 6E**). Meanwhile, NPC tissues showed increased TG(36:0) levels (**Figure 6L**). The metabolic flux from DG(44:9) to PC(20:4) is controlled by CEPT1 (**Figure 6M**), whereas DGAT2 catalyzes the conversion of DG(44:9) to triacylglycerol TG(36:0). Consistently, DGAT2 was significantly upregulated in NPC samples (**Figure 6N**), supporting a regulatory association between DGAT2 overexpression and TG(36:0) accumulation in NPC.

### Expression patterns of glycerophospholipid-related genes and metabolites across different regions and trajectory inference

**Figure 7:**
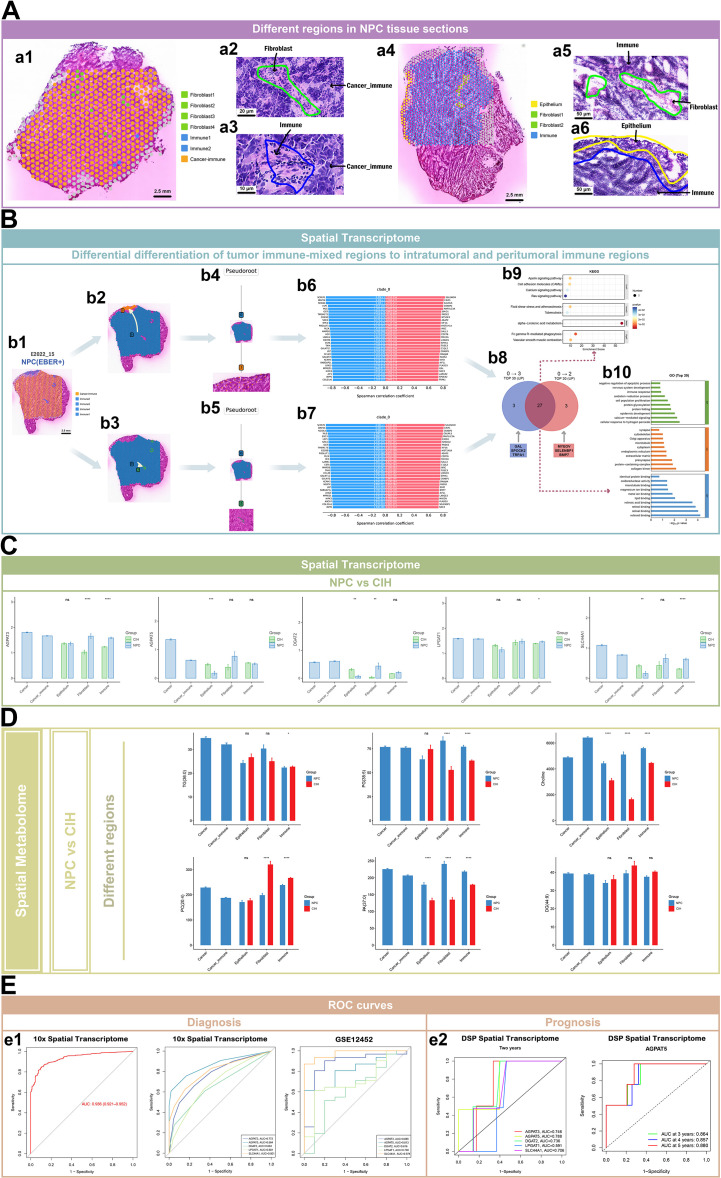
Expression patterns of glycerophospholipid-related genes and metabolites across regions, along with trajectory inference. **(A)** HE-stained tissue sections in 10x spatial transcriptomics, with fibroblast, immune cell, tumor-immune mixed, and epithelial regions color-coded. **(B)** stLearn pseudotime analysis: b1 shows the immune region (blue) and cancer-immune mixed region (orange). b2-b3 illustrates differentiation from the tumor-immune mixed region towards the peritumoral (top) and intratumoral (bottom) immune regions. b4-b5 presents dendrograms of these differentiation trajectories. b6-b7 shows changes in gene expression during differentiation, with red indicating upregulation and blue indicating downregulation. b8-b10 display GO and KEGG enrichment analyses of 27 upregulated genes during differentiation towards both peritumoral and intratumoral immune regions. **(C)** Bar plots depicting differential expression of glycerophospholipid-related genes in NPC and CIH microregions. **(D)** Bar plots illustrating glycerophospholipid-related metabolite abundance in NPC and CIH microregions. **(E)** ROC curves for combined predictions of tumor and normal epithelial regions using 5 glycerophospholipid-related genes from 10x spatial transcriptomics data (e1: left), along with individual prediction ROC curves (e1: middle). ROC curves predicting NPC vs. normal nasopharyngeal tissue using the GEO dataset (GSE12452) (e1: right). ROC curves predicting 2-year survival in NPC patients using 5 glycerophospholipid-related genes from DSP spatial transcriptomics data (e2: left), with predictions for 3, 4, and 5-year survival using AGPAT5 (e2: right).

Using HE staining and Loupe software, distinct regions of NPC and CIH tissues were annotated. The NPC/EBER- regions included fibroblast, immune, and tumor-immune mixed areas, while the CIH regions comprised epithelium, fibroblast, and immune regions (**Figure 7A**). Spatial trajectory analysis of an EBER+ NPC slice (E2022_15) revealed clear delineation between tumor and immune regions, which were further classified into intratumoral (Immune2, Immune3, and Immune4) and peritumoral (Immune1) immune regions.

Gene expression analysis along the inferred spatial trajectory from mixed tumor and immune cell regions to peritumoral immune regions showed increased expression of genes such as SELENOM, CRIP1, and CALML5. Similarly, transitions along the inferred trajectory toward intratumoral immune regions were associated with increased expression of genes including SELENOM, CRIP1, and CRABP2. Among the top 30 upregulated genes during differentiation towards both immune regions, 27 were shared; distinct genes were identified for each region: peritumoral (GAL, SPOCK2, and TRPA1) and intratumoral (MYEOV, SELENBP1, and BMP7). KEGG enrichment analysis of these 27 genes revealed significant involvement in pathways related to alpha-linolenic acid metabolism, Fc gamma R-mediated phagocytosis, vascular smooth muscle contraction, and cell adhesion molecules. Gene Ontology (GO) enrichment analysis indicated these genes participate in biological processes such as cellular response to hydrogen peroxide, cell proliferation, and apoptosis inhibition, while regulating molecular functions including lipid and metal ion binding, as well as oxidoreductase activity (**Figure 7B**).

In both fibroblast and immune regions, AGPAT3 expression was higher in NPC compared to CIH. In tumor and tumor-immune mixed regions, AGPAT3 expression was elevated in EBER+ samples relative to EBER- samples. AGPAT5 exhibited the highest expression in the tumor region, with increased expression in EBER+ versus EBER- across tumor, tumor-immune mixed, and fibroblast regions. DGAT2 expression was heightened in tumor-immune mixed regions in EBER+ compared to EBER-. In the immune region, LPGAT1 expression was greater in NPC than in CIH, and in tumor-immune mixed and fibroblast regions, LPGAT1 expression was also higher in EBER+ than EBER-. SLC44A1 showed the highest expression in the tumor region and was elevated in NPC compared to CIH in the immune region, with higher expression in EBER+ compared to EBER- in tumor, tumor-immune mixed, and fibroblast regions (**Figure 7C**, **Supplementary Figure S3A**).

In the immune region, TG(36:0) levels were higher in CIH than in NPC and elevated in EBER- compared to EBER +. PG(38:5) was more abundant in NPC than CIH in fibroblast and immune regions, and higher in EBER+ than EBER- in tumor-immune mixed regions. Conversely, in tumor, fibroblast, and immune regions, PG(38:5) was elevated in EBER- compared to EBER +. PC(20:4) was more abundant in CIH than NPC in fibroblast and immune regions, and levels were higher in EBER- than EBER+ across tumor, tumor-immune mixed, fibroblast, and immune regions. PA(37:0) levels were increased in NPC compared to CIH in epithelium, fibroblast, and immune regions, while being higher in EBER- than EBER+ in tumor and fibroblast regions. DG(44:9) levels were higher in EBER+ compared to EBER- in the epithelium region, whereas in the fibroblast region, DG(44:9) was higher in EBER- than EBER +. Choline exhibited its highest abundance in tumor-immune mixed regions, and was more abundant in NPC compared to CIH across epithelium, fibroblast, and immune regions. In the immune region, choline levels were elevated in EBER+ compared to EBER-, while in tumor, tumor-immune mixed, and fibroblast regions, choline levels were higher in EBER- than EBER+ (**Figure 7D**; **Supplementary Figure S3C**).

Using 10x spatial transcriptomics data, the combination of the five glycerophospholipid-related genes (AGPAT3, DGAT2, SLC44A1, AGPAT5, and LPGAT1) achieved an AUC of 0.936 in ROC curve analysis for predicting cancer and epithelium regions. Individually, AGPAT3, AGPAT5, and SLC44A1 predicted these regions with AUC values of 0.772, 0.864, and 0.801, respectively. Validation with an external dataset (GSE12452) confirmed that AGPAT3, AGPAT5, and SLC44A1 predicted NPC and normal nasopharyngeal tissue with AUC values of 0.865, 0.813, and 0.974, respectively (**Figure 7E** e1). In DSP spatial transcriptomics data, AGPAT3, AGPAT5, and SLC44A1 predicted the 2-year survival of NPC patients with AUC values of 0.746, 0.788, and 0.706, respectively. Furthermore, AGPAT5 successfully predicted the 3, 4, and 5-year survival rates with AUC values of 0.864, 0.857, and 0.880, respectively (**Figure 7E** e2).

### CCL11+ fibroblasts potentially modulate T cell function through metabolic reprogramming

A total of 37, 351 T and NK cells were subdivided into 14 distinct subtypes: PrecursorT, ProliferatingT_STMN1, ProliferatingT_MKI67, NK_XCL1, CD4NaiveT_CCR7, CD4NaiveT_FOS, CD4Tfh_CXCL13, CD4Treg_FOXP3, CD4Treg_IL2RA, CD4Treg_TNFRSF4, CD8NaiveT_CCR7, CD8Teff_GZMB, CD8Teff_GZMK, and CD8Teff_IFIT3 (**Supplementary Figures S4A**, **B**). A combined analysis of metabolite levels and metabolic pathway activities revealed distinct metabolic differences across T-cell subsets. Overall, the two proliferative T-cell subsets, ProliferatingT_STMN1 and ProliferatingT_MKI67, exhibited a more active metabolic state. Compared with PrecursorT cells, these proliferating T-cell populations showed an overall increase in multiple metabolites, particularly those related to carbohydrate metabolism, nucleotide biosynthesis, and amino acid metabolism. At the pathway level, ProliferatingT_STMN1 and ProliferatingT_MKI67 displayed higher activities in nucleotide metabolism, carbohydrate metabolism, and several amino acid metabolic pathways, suggesting a metabolic profile consistent with increased proliferative and biosynthetic activity. In contrast, PrecursorT cells exhibited relatively lower overall metabolic activity, with most metabolites and associated metabolic pathways present at reduced levels, reflecting a comparatively metabolically quiescent state. These findings indicate that as T cells transition from a precursor state to a proliferative state, their metabolic program shifts from relative quiescence to metabolic reprogramming characterized by increased energy supply and biosynthetic capacity (**Supplementary Figures S4C**, **D**).

To further investigate whether CCL11+ fibroblasts may influence T-cell functional states, we next analyzed the cell-cell communication between CCL11+ fibroblasts and T-cell subsets. As shown in **Supplementary Figure S5**, CCL11+ fibroblasts exhibited significant chemokine-mediated interactions with multiple T-cell populations, including PrecursorT, ProliferatingT_STMN1, and ProliferatingT_MKI67 cells. Several ligand-receptor pairs were enriched in these interactions, such as CXCL9/10/11-CXCR3 and CXCL12/14-CXCR4, indicating active communication between fibroblasts and T cells within the tumor microenvironment. Notably, the interacting T-cell subsets, particularly the proliferating T-cell populations, displayed pronounced metabolic reprogramming (**Supplementary Figures S4C**, **D**), suggesting a potential association between CCL11+ fibroblasts, chemokine-mediated signaling, and metabolic states of T-cell subsets.

## Discussion

Through single-cell transcriptome analysis, we found that fibroblasts in NPC exhibit significantly higher scores in angiogenesis, glycolysis, apoptosis, oxidative phosphorylation, and hypoxia compared to those in CIH. Notably, fatty acid scores were highest in fibroblasts relative to the other 25 cell clusters analyzed. In the spatial transcriptome, fatty acid scores in NPC surpassed those in CIH, and the spatial metabolome revealed a higher abundance of ten different fatty acids in NPC. Fibroblasts exhibiting high expression of CCL11 accumulated fatty acids, with their abundance markedly elevated in NPC compared to CIH. Spatial analysis indicated that fibroblasts are positioned closer to endothelial and malignant epithelial cells in NPC. Notably, fibroblasts with elevated CCL11 expression interacted more actively with malignant epithelial cells than with normal epithelial cells.

In the NPC microenvironment, CCL11-expressing fibroblasts engage with malignant epithelial cells through various receptor-ligand interactions, including CCL11-ACKR4, CCL11-CCR3, CCL11-CCR2, CCL11-ACKR2, and CCL11-DPP4 pairs. These interactions may contribute to malignant metabolic processes, enhanced hypoxia, and angiogenesis. Furthermore, it is plausible that CCL11-positive fibroblasts regulate fatty acid metabolism in malignant epithelial cells.

Chemokines have increasingly been recognized as regulators of metabolic reprogramming in the tumor microenvironment. CCL11 primarily signals through its canonical receptor CCR3 and can activate downstream signaling pathways such as PI3K-AKT and MAPK cascades, which are closely associated with lipid biosynthesis and fatty acid metabolism in cancer cells (25). Activation of the PI3K-AKT pathway can promote lipid metabolic reprogramming by stimulating sterol regulatory element-binding proteins (SREBPs), key transcription factors that regulate genes involved in fatty acid and phospholipid synthesis (26). Increased SREBP activity has been shown to enhance membrane lipid production and glycerophospholipid synthesis to support rapid tumor cell proliferation and metabolic adaptation (27). CCR3-mediated CCL11 signaling may further intersect with key metabolic regulators such as mTOR and PPARgamma, thereby providing a potential mechanistic link to lipid metabolic remodeling. Activation of AKT can stimulate mTORC1 and promote lipid biosynthesis through SREBP1 and downstream enzymes including AGPAT family members and DGAT2 (26, 28, 29). Meanwhile, PPARgamma may cooperate with SREBP to enhance lipid metabolic programs (30, 31). Although ACKR4 is considered an atypical chemokine receptor, it may, similar to other atypical chemokine receptors, regulate chemokine gradients and CCR3 signaling intensity within the tumor microenvironment (32). In this context, the ligand-receptor interactions identified between CCL11-expressing fibroblasts and malignant epithelial cells may contribute to lipid metabolic remodeling in NPC, potentially facilitating fatty acid accumulation and glycerophospholipid biosynthesis within tumor regions. Although our multi-omics analysis provides spatial and transcriptomic evidence supporting this hypothesis, further functional studies will be required to directly determine whether CCL11 signaling regulates glycerophospholipid metabolism in NPC cells.

Our spatial metabolomics analysis highlighted that the differential metabolites between NPC and CIH, as well as between EBER+ and EBER- groups, are significantly enriched in the glycerophospholipid metabolism pathway. Glycerophospholipids, critical components of biological membranes, are often elevated in tumors, particularly in aggressive cancers like NPC. The upregulation of fatty acids, phospholipids, and cholesterol in such tumors is well documented, with fatty acid synthase activity linked to enhanced glycerophospholipid synthesis (33). Prior research has implicated the glycerophospholipid metabolism pathway in various cancers, including esophageal squamous cell carcinoma (34), gastric cancer (35), and breast cancer (36), while its dysregulation has been associated with colorectal cancer (37), and prostate cancer (38). In head and neck squamous cell carcinoma (HNSCC), specific glycerophospholipid species, such as PC(32:1) and PC(34:1), accumulate in tumor regions (39). Our study provides evidence for the diagnostic and prognostic significance of glycerophospholipid metabolism in NPC.

Through multi-omics analysis, we identified five glycero-phospholipid-related genes—AGPAT3, DGAT2, SLC44A1, AGPAT5, and LPGAT1—exhibiting high expression in NPC and EBER+ tumor-enriched regions. The elevated expression of these genes correlates with poorer prognosis in NPC patients, reinforcing their potential role in cancer progression. For instance, the activation of the MEK/ERK/SRF axis has been shown to upregulate AGPS and AGPAT3, enhancing susceptibility to ferroptosis in gastric cancer cells (40). Given the association between ferroptosis and NPC prognosis (41), targeting this pathway could offer therapeutic potential. However, it is essential to note that EBV infection may diminish NPC cell sensitivity to ferroptosis by activating the p62-Keap1-NRF2 signaling pathway and upregulating SLC7A11 and GPX4 (42). AGPAT5 displays a dual role in cancer; it acts as a tumor suppressor in colorectal cancer, correlating with improved survival outcomes (43), while in hepatocellular carcinoma (HCC), higher AGPAT5 expression is linked to poorer prognosis (44). Our findings in NPC align with the latter, suggesting that AGPAT5 may contribute to adverse outcomes in this context. Additionally, SLC44A1 serves as a prognostic marker for head and neck carcinomas (45), reinforcing its relevance in NPC. DGAT2 is also upregulated in several cancers, including bladder, breast, and thyroid cancers (46), and its overexpression in HCC has been linked to reduced cell proliferation by facilitating triglyceride synthesis (46, 47). LPGAT1’s overexpression correlates with poor prognosis in lung adenocarcinoma and colorectal cancer (48, 49), consistent with our observations in NPC. The identification of AGPAT3, DGAT2, SLC44A1, AGPAT5, and LPGAT1 may provide important opportunities for improving NPC diagnosis and prognosis assessment. The elevated expression of these glycerophospholipid metabolism-related genes in tumor-enriched regions suggests that they could potentially be detected in tumor biopsy samples through transcriptomic profiling or immunohistochemical analysis, and may serve as candidate biomarkers for identifying metabolically active tumor subtypes. In addition, integrating these genes into multi-gene prognostic models may help stratify NPC patients according to metabolic risk, thereby supporting more precise clinical management. Although the five-gene signature demonstrated strong diagnostic performance, its prognostic value was limited in this study. The multivariate Cox regression analysis did not reach statistical significance, possibly due to the relatively small sample size. Therefore, its utility for survival prediction requires further validation in larger cohorts. Importantly, enzymes involved in glycerophospholipid metabolism are increasingly recognized as potential therapeutic targets, as disrupting lipid biosynthesis and membrane remodeling can impair tumor growth and metabolic adaptation. Previous studies have shown that targeting lipid metabolic pathways can inhibit cancer cell proliferation and enhance sensitivity to anticancer therapies (27, 50, 51). Therefore, therapeutic strategies aimed at modulating glycerophospholipid metabolism, including inhibition of enzymes such as AGPAT family members or DGAT2, may represent a promising direction for future NPC treatment.

The spatial trajectory analysis in NPC slices indicated that genes upregulated during differentiation from the cancer-immune mixed region to both peritumoral and intratumoral immune regions largely overlap, suggesting shared mechanisms driving NPC invasion. Key cellular processes involved include cell proliferation, apoptosis inhibition, cell adhesion, and metabolic pathways. Notably, there is significant heterogeneity in NPC invasion; for instance, GAL, SPOCK2, and TRPA1 were specifically upregulated during differentiation toward the peritumoral immune region, while MYEOV, SELENBP1, and BMP7 were elevated during differentiation toward the intratumoral immune region.

Despite these findings, several limitations of this study should be acknowledged. First, the number of samples used for single-cell transcriptomics, spatial transcriptomics, and spatial metabolomics analyses was relatively limited, which may restrict the generalizability of our findings. Future studies with larger independent cohorts will be required to further validate the robustness of the observed molecular and metabolic patterns. Second, although our multi-omics integration revealed spatial associations between CCL11-expressing fibroblasts, fatty acid accumulation, and glycerophospholipid metabolism in malignant epithelial cells, these observations are primarily based on computational inference and correlative evidence. Additional mechanistic experiments, such as *in vitro* and *in vivo* functional studies, will be necessary to directly determine the regulatory role of CCL11 signaling in lipid metabolic reprogramming in NPC. Specifically, functional experiments such as fibroblast-NPC co-culture systems, CCL11 knockdown or neutralization, and CCR3 inhibition assays combined with lipid accumulation assessment would be valuable to directly validate the CCL11-CCR3 axis in regulating lipid metabolic reprogramming. Third, while the prognostic value of the identified glycerophospholipid metabolism-related genes was supported by transcriptomic analyses, further clinical validation in larger prospective cohorts and protein-level validation will be needed to confirm their clinical applicability. Fourth, for spatial pseudotime inference, we used the algorithm’s default branch confidence score provided by stLearn to support trajectory reconstruction. However, independent validation using orthogonal molecular markers (e.g., spatially resolved marker gene dynamics or pathway-specific signatures across trajectory branches) was not fully performed. Incorporating such independent validation would further strengthen the robustness and biological interpretability of the inferred spatial trajectories. Finally, although EBER+ tissues showed higher immune diversity and fibroblast interactions, the limited number of EBER- samples means that future studies with larger cohorts are needed to validate these findings.

In conclusion, by integrating single-cell transcriptomics, 10x spatial transcriptomics, and spatial metabolomics, we constructed a comprehensive spatial cell atlas, gene expression profile, and metabolite distribution for NPC and CIH. Our findings suggest that fibroblasts may play an important role in malignant metabolism within the NPC microenvironment, potentially contributing to hypoxia and angiogenesis. The accumulation of fatty acids in NPC is associated with CCL11-expressing fibroblasts, which are spatially proximate to endothelial and malignant epithelial cells. The interactions mediated by CCL11 highlight a potential mechanism that may be involved in fibroblast-mediated tumor progression. Our study delineates the differentiation trajectories from the cancer-immune mixed region to both the peritumoral and intratumoral immune regions in NPC, revealing both commonalities and heterogeneity in the progression and spatial organization of NPC. The glycerophospholipid biosynthesis pathway emerges as a pivotal player in NPC pathology. Moreover, the identification of AGPAT3, DGAT2, SLC44A1, AGPAT5, and LPGAT1 as potential diagnostic and prognostic markers for NPC may provide new insights for therapeutic intervention and personalized treatment strategies.

## Data Availability

The raw DSP spatial transcriptomic data have been deposited in the Genome Sequence Archive (GSA) of the National Genomics Data Center (accession: HRA003609) and are publicly available at https://ngdc.cncb.ac.cn/gsa-human/browse/HRA003609. The single-cell sequencing data reported in this paper have been deposited in OMIX at the China National Center for Bioinformation (accession: OMIX013224) and are available at https://ngdc.cncb.ac.cn/omix/release/OMIX013224. The original contributions presented in the study are included in the article. Further inquiries can be directed to the corresponding author on reasonable request.

## Supplementary material

Supplementary Figure 1 Analysis of Top2 cell type in each spatial section and metabolite screening. **(A)** The cell type with the second highest proportion in each spot (Top2 type). If the Top1 cell type comprises over 70% in any spot, Top2 will be replaced by Top1. Different colors represent distinct cell types. **(B)** Bar plot showing the average abundance of the top 10 accumulated and depleted metabolites in Fibroblasts_MMP1, Fibroblasts_MMP11, and Fibroblasts_MKI67 across groups. **(C)** Top 10 upregulated and downregulated metabolites with the highest VIP scores in EBER+ NPC vs. EBER- NPC, where larger dots indicate greater VIP values (left). Box plots of the top 10 significantly different metabolites ranked by VIP in EBER+ NPC vs. EBER- NPC (right).

Supplementary Figure 2 Transcription factors in fibroblast clusters and spatial co-localization of malignant epithelial cells. **(A)** The top 5 regulons of Fibroblasts_MMP1, Fibroblasts_MMP11, and Fibroblasts_MKI67 in NPC and CIH. **(B)** Euclidean distances of Malignant Epithelial Cells from surrounding cells in EBER+ NPC (b1) and EBER- NPC (b2), represented from red (near) to blue (far) (b1-b2: top left). Distances are categorized into two or three levels: reference (0), start (at the 1/3 quantile distance), and end (farthest away) (b1-b2: top right). Density plots of cell types at varying distances from Malignant Epithelial Cells, with colors representing different cell types (b1-b2: bottom). **(C)** Differential gene analysis between NPC and CIH (left) and EBER+ NPC vs. EBER- NPC (right), with upregulated genes depicted in red and downregulated genes in blue.

Supplementary Figure 3 Impact of EBER on glycerophospholipid-related genes and metabolites across regions. **(A)** Bar plots comparing the expression of glycerophospholipid-related genes between EBER+ and EBER- microregions. **(B)** Multivariate Cox regression analysis of the five-gene signature and clinical variables. **(C)** Violin plots depicting the abundance of glycerophospholipid-related metabolites in EBER+ and EBER- regions.

Supplementary Figure 4 Metabolic characteristics of T and NK cell subsets. **(A)** UMAP plot showing the distribution of T and NK cell subsets. **(B)** Violin plot illustrating the expression of representative marker genes across T and NK cell subsets. **(C)** Heatmap showing the relative abundance of metabolites among different T and NK cell subsets. **(D)** Heatmap depicting the activity of metabolic pathways across T and NK cell subsets.

Supplementary Figure 5 Cell-cell communication between CCL11+ fibroblasts and T-cell subsets (PrecursorT, ProliferatingT_STMN1 and ProliferatingT_MKI67). Dot size represents the mean interaction strength, and color indicates the statistical significance.
