# Supplementary figures and images for: Exploring glycerophospholipid metabolism in nasopharyngeal carcinoma: interactions between malignant epithelial cells and CCL11-expressing fibroblasts
Source: Front Immunol. 2026 May 20;17:1799551. doi: 10.3389/fimmu.2026.1799551 (PMC13229993; doi:10.3389/fimmu.2026.1799551)

A

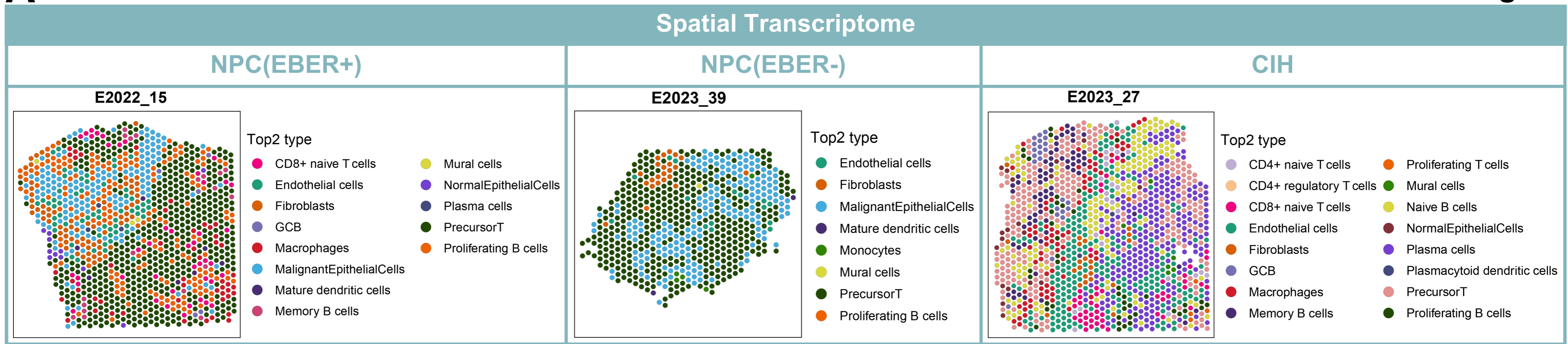

B

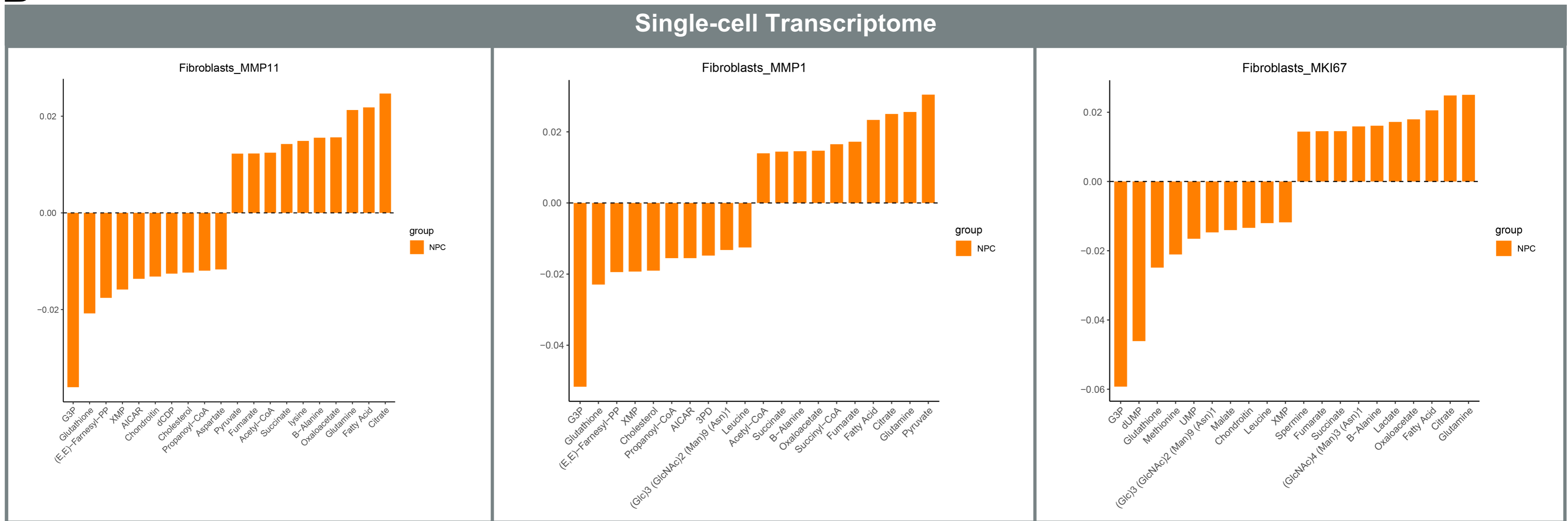

C

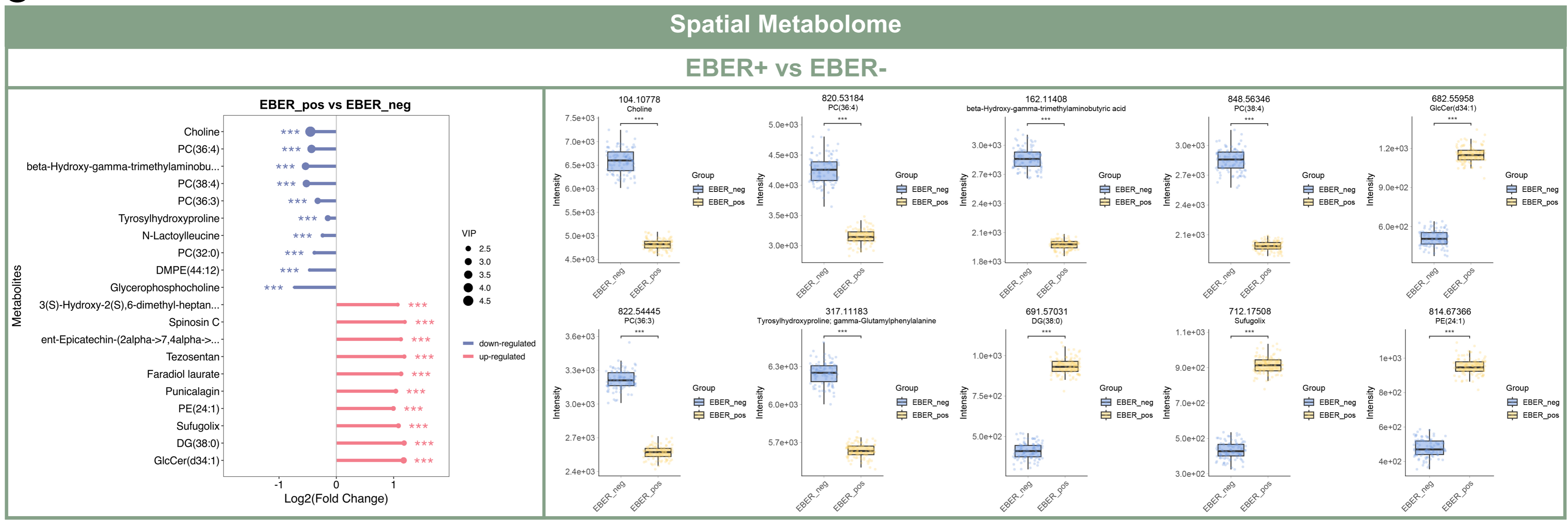

Supplement: Supplementary Figure 1 — Analysis of Top2 cell type in each spatial section and metabolite screening. (A) The cell type with the second highest proportion in each spot (Top2 type). If the Top1 cell type comprises over 70% in any spot, Top2 will be replaced by Top1. Different colors represent distinct cell types. (B) Bar plot showing the average abundance of the top 10 accumulated and depleted metabolites in Fibroblasts_MMP1, Fibroblasts_MMP11, and Fibroblasts_MKI67 across groups. (C) Top 10 upregulated and downregulated metabolites with the highest VIP scores in EBER+ NPC vs. EBER- NPC, where larger dots indicate greater VIP values (left). Box plots of the top 10 significantly different metabolites ranked by VIP in EBER+ NPC vs. EBER- NPC (right). [file Image1.pdf]

A

## Single-cell Transcriptome

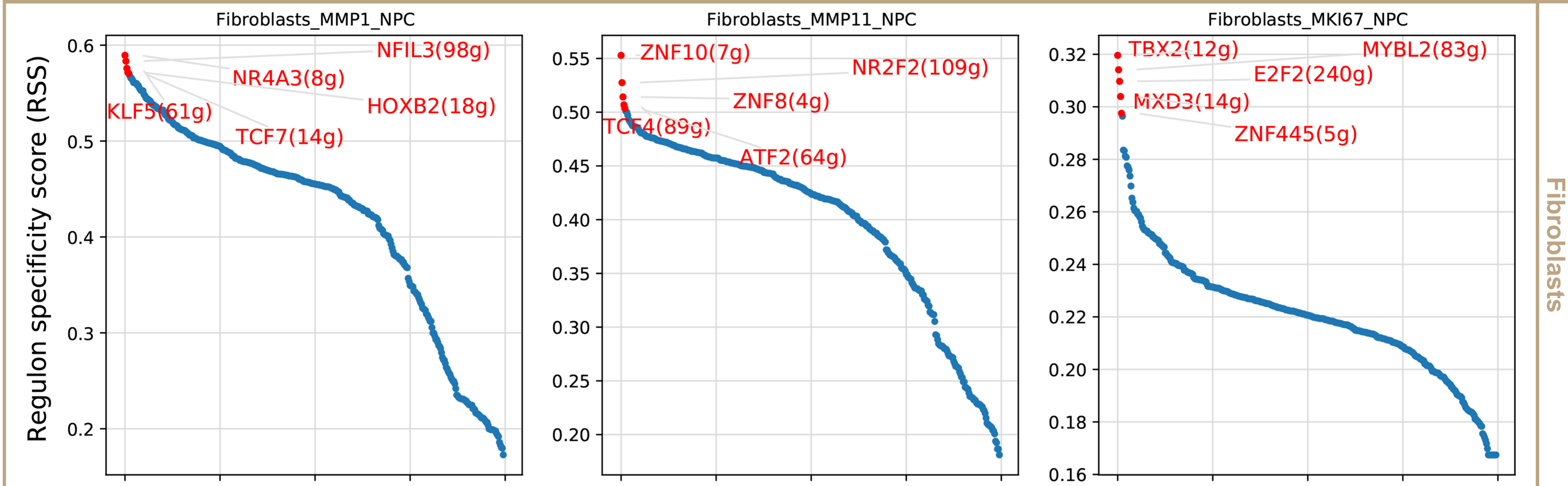

B

## Spatial Transcriptome

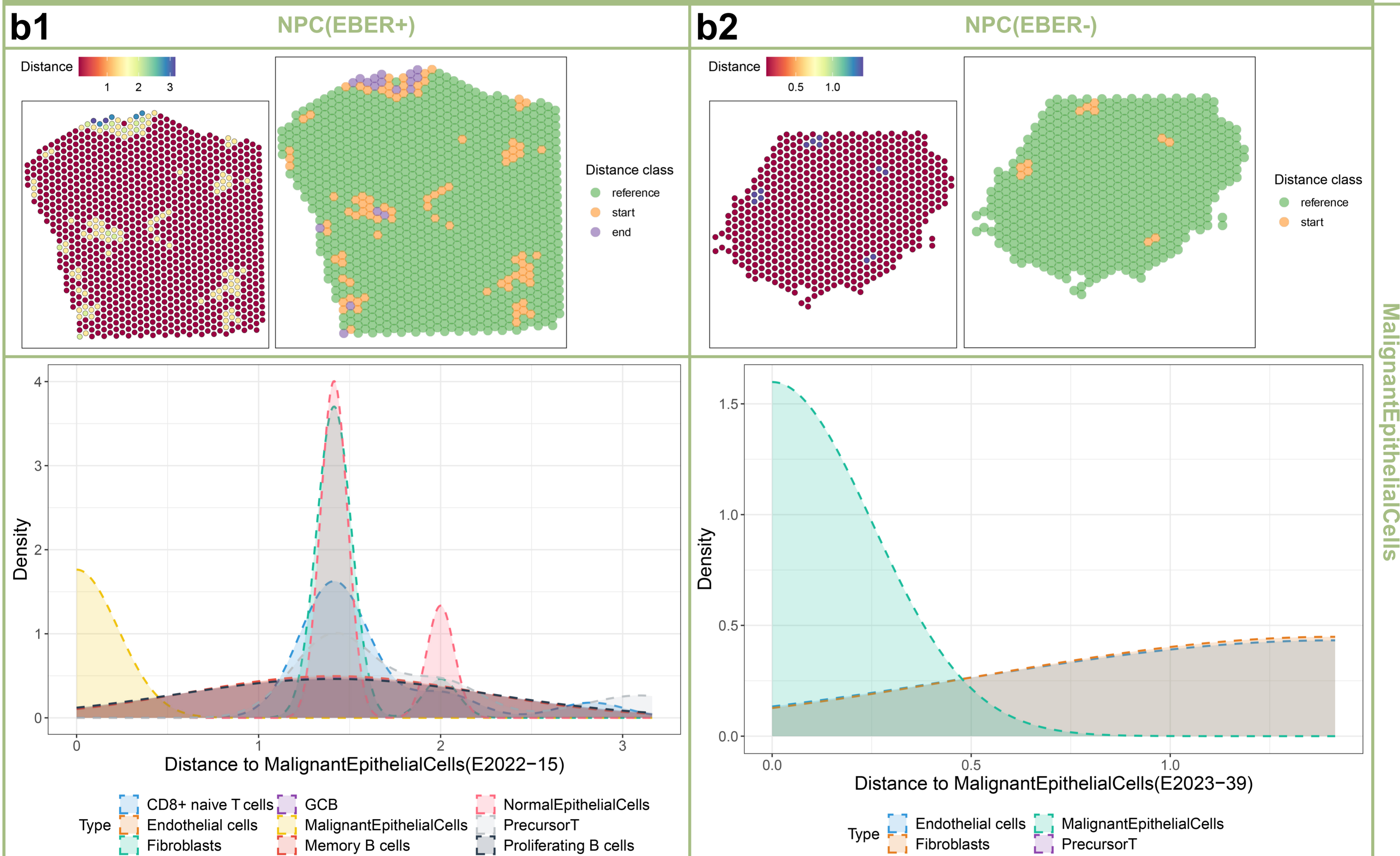

C

## Spatial Transcriptome

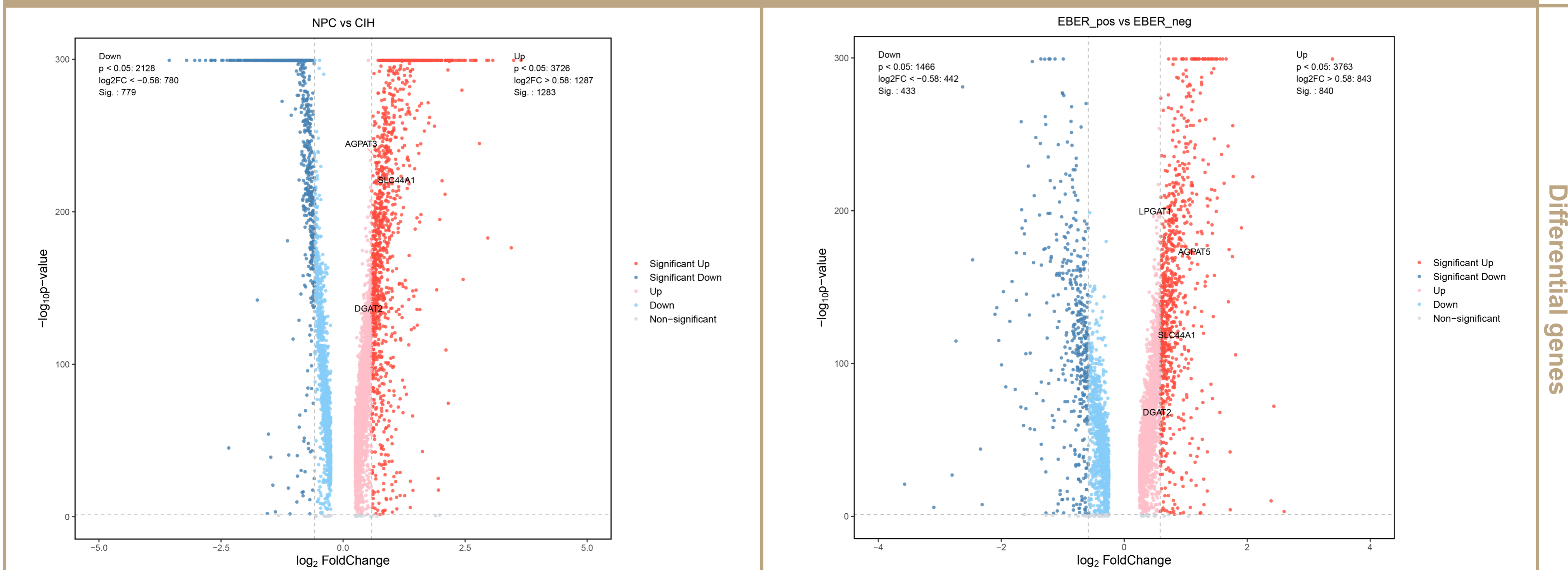

Supplement: Supplementary Figure 2 — Transcription factors in fibroblast clusters and spatial co-localization of malignant epithelial cells. (A) The top 5 regulons of Fibroblasts_MMP1, Fibroblasts_MMP11, and Fibroblasts_MKI67 in NPC and CIH. (B) Euclidean distances of Malignant Epithelial Cells from surrounding cells in EBER+ NPC (b1) and EBER- NPC (b2), represented from red (near) to blue (far) (b1-b2: top left). Distances are categorized into two or three levels: reference (0), start (at the 1/3 quantile distance), and end (farthest away) (b1-b2: top right). Density plots of cell types at varying distances from Malignant Epithelial Cells, with colors representing different cell types (b1-b2: bottom). (C) Differential gene analysis between NPC and CIH (left) and EBER+ NPC vs. EBER- NPC (right), with upregulated genes depicted in red and downregulated genes in blue. [file Image2.pdf]

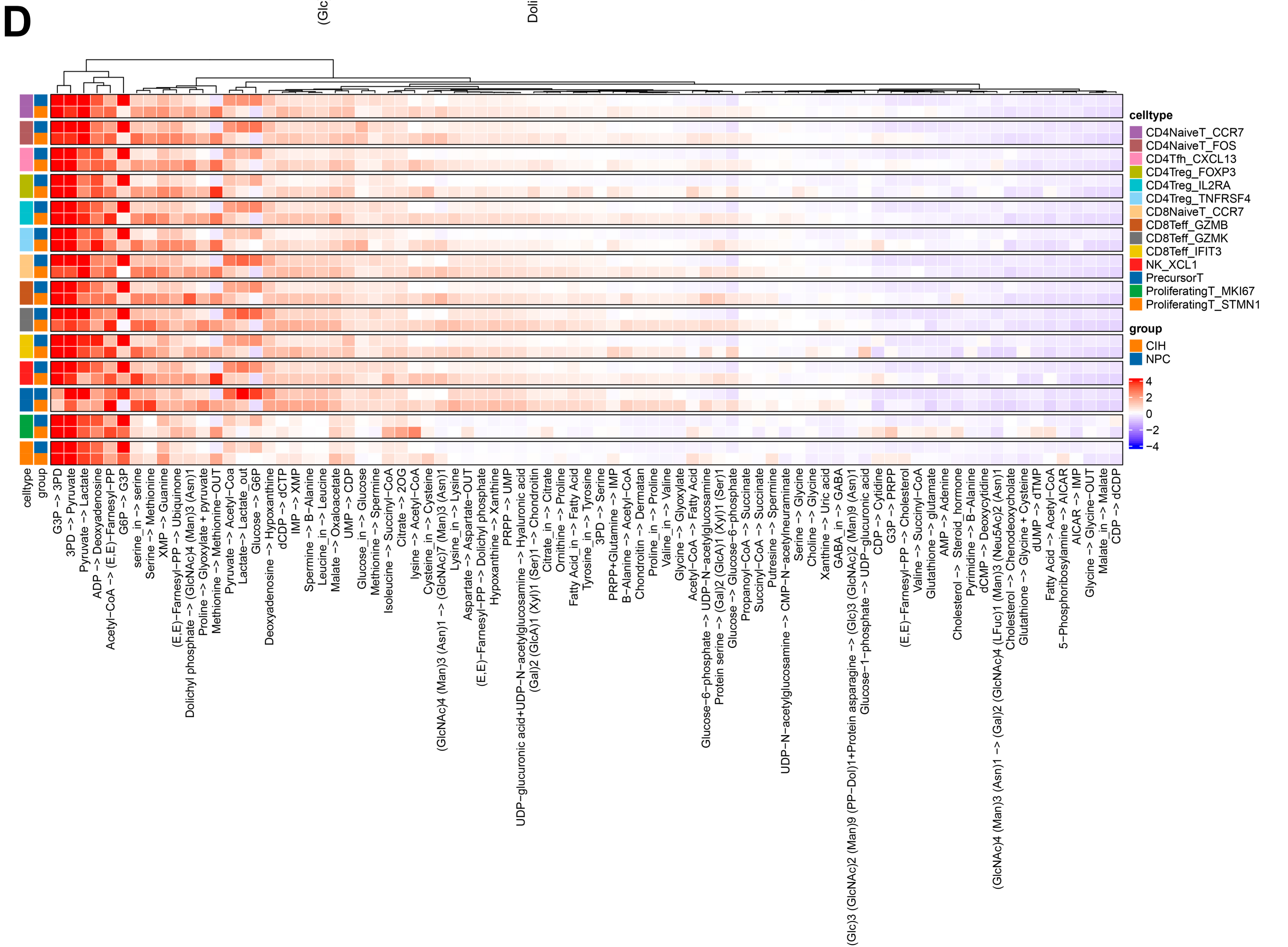

Supplement: Supplementary Figure 4 — Metabolic characteristics of T and NK cell subsets. (A) UMAP plot showing the distribution of T and NK cell subsets. (B) Violin plot illustrating the expression of representative marker genes across T and NK cell subsets. (C) Heatmap showing the relative abundance of metabolites among different T and NK cell subsets. (D) Heatmap depicting the activity of metabolic pathways across T and NK cell subsets. [file Image4.pdf]

Single-cell Transcriptome

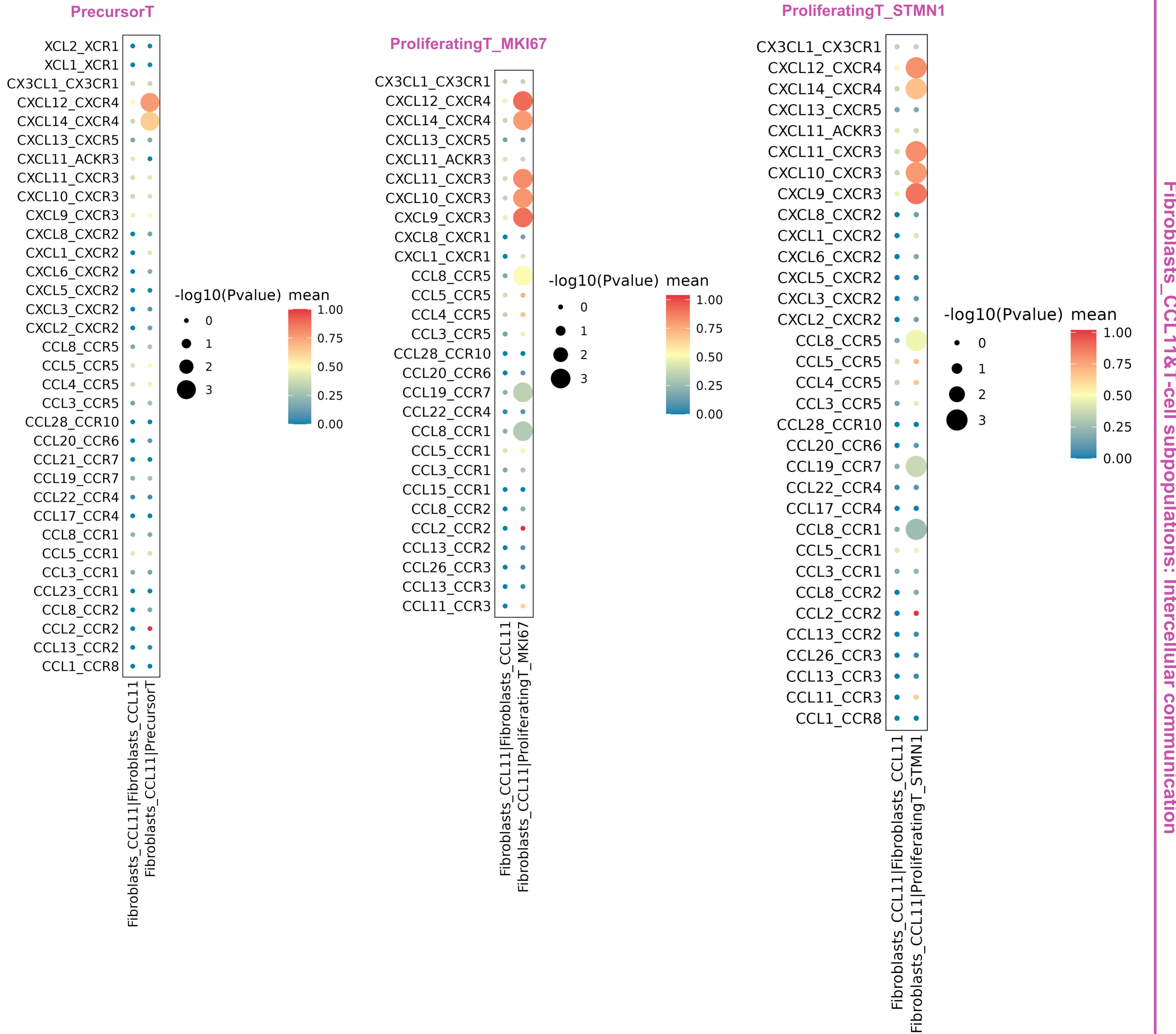

Supplement: Supplementary Figure 5 — Cell-cell communication between CCL11+ fibroblasts and T-cell subsets (PrecursorT, ProliferatingT_STMN1 and ProliferatingT_MKI67). Dot size represents the mean interaction strength, and color indicates the statistical significance. [file Image5.pdf]
