# Supplementary material for: Exploring glycerophospholipid metabolism in nasopharyngeal carcinoma: interactions between malignant epithelial cells and CCL11-expressing fibroblasts
Source: Front Immunol. 2026 May 20;17:1799551. doi: 10.3389/fimmu.2026.1799551 (PMC13229993; doi:10.3389/fimmu.2026.1799551)
Supplement: Supplementary Figure 3 — Impact of EBER on glycerophospholipid-related genes and metabolites across regions. (A) Bar plots comparing the expression of glycerophospholipid-related genes between EBER+ and EBER- microregions. (B) Multivariate Cox regression analysis of the five-gene signature and clinical variables. (C) Violin plots depicting the abundance of glycerophospholipid-related metabolites in EBER+ and EBER- regions. [file Image3.pdf]

Fig. s3

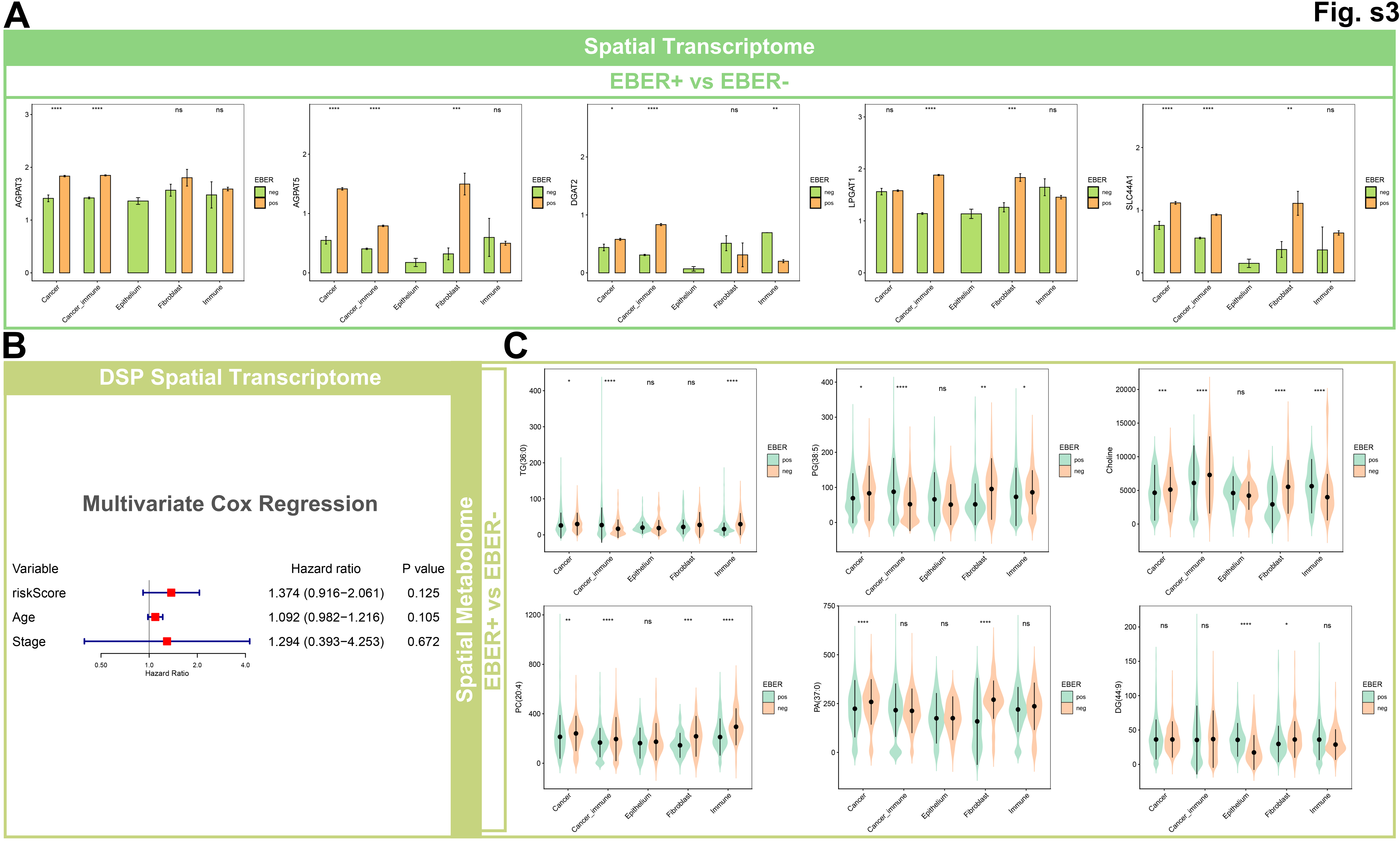
A
Spatial Transcriptome
EBER+ vs EBER-
AGPAT3
AGPAT5
DGAT2
LPGAT1
SLC44A1
EBER
neg
pos
Cancer
Cancer_immune
Epithelium
Fibroblast
Immune
B
DSP Spatial Transcriptome
Multivariate Cox Regression
Variable | Hazard ratio | P value
riskScore | 1.374 (0.916−2.061) | 0.125
Age | 1.092 (0.982−1.216) | 0.105
Stage | 1.294 (0.393−4.253) | 0.672
0.50 1.0 2.0 4.0
Hazard Ratio
C
Spatial Metabolome
EBER+ vs EBER-
TG(36:0)
PG(38:5)
Choline
PC(20:4)
PA(37:0)
DG(44:9)
EBER
pos
neg
